# Assessment of the predictive capacity of a physiologically based kinetic model using a read-across approach

**DOI:** 10.1016/j.comtox.2021.100159

**Published:** 2021-05

**Authors:** Alicia Paini, Andrew Worth, Sunil Kulkarni, David Ebbrell, Judith Madden

**Affiliations:** a European Commission, Joint Research Centre (JRC), Ispra, Italy; b Existing Substances Risk Assessment Bureau, Health Canada, Ottawa, Canada; c School of Pharmacy and Biomolecular Sciences, Liverpool John Moores University, Byrom Street, Liverpool L3 3AF, UK

**Keywords:** PBK model, Risk assessment, Read-across, Analogues, Kinetics

## Abstract

With current progress in science, there is growing interest in developing and applying Physiologically Based Kinetic (PBK) models in chemical risk assessment, as knowledge of internal exposure to chemicals is critical to understanding potential effects in vivo. In particular, a new generation of PBK models is being developed in which the model parameters are derived from in silico and in vitro methods. To increase the acceptance and use of these “Next Generation PBK models”, there is a need to demonstrate their validity. However, this is challenging in the case of data-poor chemicals that are lacking in kinetic data and for which predictive capacity cannot, therefore, be assessed. The aim of this work is to lay down the fundamental steps in using a read across framework to inform modellers and risk assessors on how to develop, or evaluate, PBK models for chemicals without in vivo kinetic data. The application of a PBK model that takes into account the absorption, distribution, metabolism and excretion characteristics of the chemical reduces the uncertainties in the biokinetics and biotransformation of the chemical of interest. A strategic flow-charting application, proposed herein, allows users to identify the minimum information to perform a read-across from a data-rich chemical to its data-poor analogue(s). The workflow analysis is illustrated by means of a real case study using the alkenylbenzene class of chemicals, showing the reliability and potential of this approach. It was demonstrated that a consistent quantitative relationship between model simulations could be achieved using models for estragole and safrole (source chemicals) when applied to methyleugenol (target chemical). When the PBK model code for the source chemicals was adapted to utilise input values relevant to the target chemical, simulation was consistent between the models. The resulting PBK model for methyleugenol was further evaluated by comparing the results to an existing, published model for methyleugenol, providing further evidence that the approach was successful. This can be considered as a “read-across” approach, enabling a valid PBK model to be derived to aid the assessment of a data poor chemical.

## Highlights

• Potential regulatory application of PBK modelling information to assist read-across.
• Presents workflow to read across PBK model information from data-rich to data-poor chemicals.
• Describes appropriate analogue selection based on a set of specific criteria.
• Uses estragole and safrole as source chemicals for a target chemical - methyleugenol.
• Example of PBK model validation where in vivo kinetic data are lacking.

## 1 Introduction

Internal dose metrics are considered more predictive of biological responses than external doses when assessing and managing risks of chemicals to human health and the environment [1]. Physiologically Based Kinetic (PBK) models are mathematical models based on ordinary differential equations, which can be used to predict internal dose metrics by taking into account the physicochemical properties of the substance of interest along with the physiological and biochemical processes in a species of interest. These processes determine the fate of a chemical in an organism by means of its absorption, distribution, metabolism and excretion (ADME) characteristics. A PBK model includes both chemical-independent parameters (anatomical and physiological), as well as chemical-dependent parameters (physicochemical and ADME properties). However, the regulatory uptake of these models has been slow, due to lack of understanding and/or trust in the models [2], [3].

With current progress in science and risk assessment, there is growing interest in developing, reporting, evaluating and applying PBK models, accompanied by a shift towards next generation PBK models. The difference between traditional and next generation PBK models is as follows; on the one hand, a traditional PBK model is calibrated and evaluated using in vivo data - the model structure reflects a balance between the principles of parsimony (minimal but essential elements characterising the system) and plausibility (reflective of physiological reality and consistent with the current state of knowledge). A “familiar uncertainty” based on the conceptual model parameters used, as well as the dose metrics applied is present in the traditional model (for example uncertainty arising from inter- and intra-species biological variability). On the other hand, a next generation PBK model is developed relying on in vitro or in silico methods. Model structure reflects a more detailed mechanistic understanding of biology and biochemistry, but this is accompanied by more “unfamiliar uncertainties” (for example, uncertainty relating to the relevance, reliability and variability of the in vitro and in silico methods from which model parameters are generated). These next generation PBK models, in the ideal situation, promise increased predictive potential, as well as mechanistic insights due to inclusion of mechanistic processes and emerging (human-relevant) data, but introduce additional challenges for risk assessors attempting to review and use these more detailed and complex models in support of regulatory decision making. This is especially true where experimental in vivo kinetic data are not available for comparison and evaluation of the model predictions. Conversely, in current practice, where PBK models are developed without incorporating mechanistic knowledge, these are associated with greater uncertainty and do not have the potential to provide improved predictivity or mechanistic insights; hence next generation PBK models have distinct advantages. To address the lack of uptake of PBK models by the regulatory sector, a group of experts in the field proposed a way forward for model evaluation, establishing a list of elements that could be used to assess the validity of next generation PBK models [3]. Among these elements the read-across approach was proposed and is further illustrated here. This has also been described in the recently published Organisation for Economic Cooperation and Development (OECD) PBK model guidance document [4].

As introduced above, traditional model parameterisation, calibration and validation relies heavily on comparing model simulations with in vivo data i.e. blood/plasma or tissue concentrations. The availability of in vivo kinetic data is limited to a few well-studied (data-rich) chemicals and only for certain species of interest. This impedes the validation and use, in a regulatory context, of PBK models for chemicals that lack such data. Therefore, for those chemicals without toxicokinetic (TK) information (data-poor), other lines of evidence are required to evaluate the suitability of PBK models for the intended purposes.

Read across, one such line of evidence, is a technique for predicting endpoint information for one substance (the target substance) by using data for the same endpoint from (an)other substance(s), referred to as (a) source substance(s) [5]. This approach is increasingly being applied for data gap filling in chemical hazard assessment. In the case of PBK modelling, for those cases in which in vivo data exist for one chemical, a read-across approach may be applied to parameterise models for other similar chemicals [6], [7], [8], [9]. For example, if a valid PBK model exists for chemical A (source chemical), whereas chemical B (target chemical) lacks any in vivo data, but has been shown to be similar in structure, and/or other relevant properties, to chemical A, then the same parameterised PBK model structure/code and in vivo data for chemical A may be suitable to derive a model for chemical B. Alternatively, if parameterisation of the PBK model using available in vitro or in silico data for chemical B is possible, predictions can be compared to the output from the model for chemical A based on in vivo data, in order to evaluate the PBK model for chemical B. When using such a model based on similarity between different chemicals, the influence of chemical-specific properties mediating ADME behaviour (such as logarithm of the octanol:water partition coefficient (log P/ log Kow), presence of specific functional groups etc) should also be carefully considered. As mentioned above, developing PBK models for data-poor chemicals relies on in vitro and in silico methods for deriving ADME relevant parameters. Therefore, there is an increasing need for a more systematic characterisation of these alternative methods for evaluating ADME predictions without the use of in vivo data. Ellison et al. report one of the first attempts in this direction, testing the hypothesis that an adequately developed PBK model for a target chemical (chemical with no in vivo kinetic data) can be evaluated using PK data from a source chemical (chemical with existing in vivo kinetic data) [7]. These authors compared PK profiles and model simulations from PBK models that were developed using in vivo data. Recently the PK analogue approach was applied to a PBK model built only on in vitro and in silico data, using caffeine and diphenhydramine as examples [8].

In 2016 Lu et al. published a Knowledgebase of 307 chemicals for which existing PBK models were available [6]. The authors demonstrated that it was possible to use an existing PBK model for one chemical (the source chemical) to inform the development of a PBK model for a similar (target) chemical, by adjusting chemical-specific model parameters. No chemical can be considered as being absolutely similar to another, only similar in terms of specific properties; Lu et al. established similarity on the basis of physico-chemical properties. The approach described here can be considered as a read-across approach for PBK models where a data-rich analogue is used to infer information for a data-poor chemical; as with all read-across approaches, it is essential to fully justify the choice of the analogue(s).

Similarity can be considered in terms of structural similarity (using fingerprint methods) physico-chemical properties, functional/mechanism of action or metabolite similarity. In this analysis, structural similarity (ascertained using fingerprints) was used to select potential analogues with existing PBK models, from which the final selection was based on expert judgement. Fingerprint methods search for specific structural features or “keys” (e.g. functional groups) within molecules. If the feature is present in a chemical structure a value of “1” is recorded; if absent “0” is recorded. In this way, bit-strings are generated comprising a series of 1 s or 0 s for the presence or absence of particular features. The number of features in common can be compared, resulting in an overall similarity score, for example a Tanimoto coefficient. These values range from 0 to 1, where 1 indicates highly similar or identical chemicals [10]. As different types and numbers of structural features are used in the different similarity assessment methods available, many different similarity scores can be generated for any given pair of chemicals; there is no consensus yet as to which method is most appropriate [11].

In this paper we describe a strategy for deriving a PBK model for a data-poor chemical using a read-across approach, which can be applied where in vivo kinetic data are not available for validation.

## 2 Methodology

### 2.1 Case study selection

The alkenylbenzene family of chemicals was selected for the present analysis as a pertinent case study. The alkenylbenzene methyleugenol was selected as the target chemical for this case study because of current interest in the carcinogenic potential of chemicals in this group and the availability of relevant toxicokinetic data. Potential analogues of methyleugenol that could be used to parameterise and evaluate a PBK model for this target chemical were selected according to the method described below.

In the following *illustrative* example methyleugenol was used as the target chemical in a proof-of-principle analysis where it was hypothetically posited there were no kinetic data to validate the PBK model. However, it is known that such information is available for this chemical, and this was used post analysis to demonstrate the validity of the approach.

### 2.2 Strategy for the identification of appropriate analogues

**Fig. 1:**
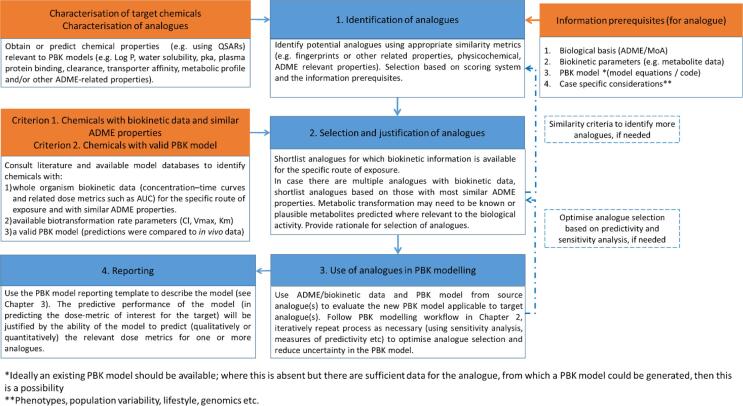
Workflow for identifying and using analogues for PBK model development and evaluation, as reported in the OECD PBK model guidance document [4].

The recently published OECD PBK model guidance document elaborates a workflow that can be used to identify analogues for PBK model development or evaluation. Analogues are sought with existing biokinetic data and/or an existing PBK model that can be used to fill data gaps in chemical safety assessment. The workflow from the OECD document is reproduced in Fig. 1. The first step in this workflow is the identification of potential analogues [4].

#### 2.2.1 Preliminary investigation of existing PBK models and identification of potential analogues

A thorough literature search for PBK models for alkylbenzenes was conducted to identify candidate analogues, using a wide range of available electronic resources [12]. The group of Professor Ivonne Rietjens in the Department of Toxicology (Wageningen, The Netherlands) is noted for investigations into many chemicals, providing in vitro biokinetic data to develop PBK models - amongst these the alkenylbenzene family are the most studied. Publications from this group were considered a good source of PBK model code for potential analogues of methyleugenol for use in this study. Potential analogues were also sought using the OECD QSAR Toolbox (https://www.oecd.org/chemicalsafety/risk-assessment/oecd-qsar-toolbox.htm; ver 4.4). In this instance the Tanimoto coefficient was used to assess the level of similarity between the target chemical and potential source analogues. A cut-off value of 0.6 was used to determine if the chemicals were structurally similar. Cut-off values for similarity are chosen arbitrarily and the chemicals identified as “similar” will be dependent on both the similarity metric and the cut-off value chosen (as discussed above). The value of 0.6 for the Tanimoto coefficient has previously been demonstrated to be a suitable cut-off for identifying toxicologically similar chemicals [13]. The mammalian metabolism simulators in the Toolbox were also used to assess the potential of analogues to form the 1′-hydroxy metabolite – relevant to the known toxicity of these chemicals.

In parallel, the Knowledgebase, developed by Lu et al., is a ready-made database of chemicals for which it is known that PBK models exist [6]. Therefore, this database was searched for any chemicals “similar” to the target methyleugenol. The Knowledgebase was supplemented with additional models that had been identified during the literature search, to ascertain which chemicals were most similar. Although it was anticipated that the models obtained specifically when searching for alkylbenzenes would be the most suitable, incorporating the more extensive Lu et al. Knowledgebase into the similarity search ensured that no existing, potentially relevant, models were overlooked. As already stated, the selection of similar chemicals depends on the similarity metric and cut-off value selected, hence, a novel workflow, developed in-house using the KNIME platform (ver 4.1.4; available on request from the authors) was used to identify potential analogues. Using the KNIME workflow, nine different fingerprints were applied (Morgan, feat Morgan, Torsion, Avalon, Layered, AtomPair, RDKit, MACCS and Pattern), the chemicals that were ranked as the top five most similar chemicals, according to each of the similarity metrics were exported. Molecular weight (MW) and the logarithm of the octanol:water partition coefficient (log Kow, as predicted by EPISUITE version 1.69) were also recorded for comparison. In this manner several potential analogues were identified.

#### 2.2.2 Strategic flowchart to assist in the selection of PBK analogues

**Fig. 2:**
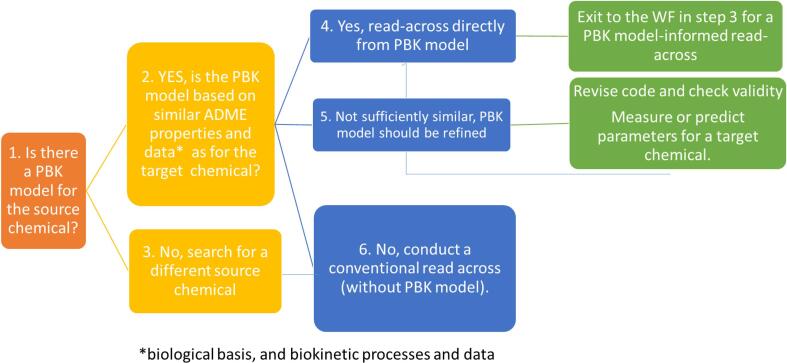
Schematic representation of the strategic decision tree to assist selection of analogues in the context of developing a PBK model for a chemical that has no in vivo data for validation.

Step 2 of the workflow in Fig. 1 is the selection of analogues. A strategic flow-chart application was developed to assist selection of suitable analogues, incorporating a decision tree (Fig. 2) that links to the workflow shown in Fig. 1. The decision tree starts by asking “is there a PBK model for your source chemical?” If yes, the PBK model for the source chemical should include a similar biological basis and biokinetic processes (ADME properties) and parameterisation to the target chemical. If this is the case, the model can be applied to make predictions using available in vitro ADME data from the target chemical, exiting the decision tree into the workflow for identifying and using analogues for PBK model development/evaluation as shown in Fig. 1 (at step 3). If the PBK model found is not sufficiently similar, the PBK model should be refined and the model code checked for validity. Finally, if in both steps the option is not possible the advice is to search for a new source chemical or conduct a conventional read across. The workflow was built for identification of analogues, enabling the user (a risk assessor) to identify the best analogue for the assessment purpose, the context of use (problem formulation) should be taken into account.

### 2.3 Using information from selected analogues for PBK model development

Using the procedures outlined in Section 2.2, estragole and safrole were selected as appropriate source chemicals for building a PBK model for the target – methyleugenol.

The source codes for the human PBK models for estragole and safrole are available as Supplementary information (appendix I). The PBK model for estragole was first published by Punt et al. [14] and revisited by Ning et al. [15]. The PBK model for safrole was published by Martati et al. [16]. The validation of both models was done using historical data from test subjects. Punt et al. [14] evaluated model performance by comparing the predicted formation of 4- allylphenol and 1′-hydroxyestragole glucuronide to literature reported levels of these metabolites in humans exposed to estragole, using information available in Sangster et al. [17]. For the evaluation of the safrole model performance, a comparison was made between the predicted total amount of urinary metabolites of safrole and the reported total levels of metabolites in the urine of humans exposed to safrole [16].

The PBK model codes were used with the following alterations:

1. The molecular weight (MW) for parent and hydroxy-metabolite (known to be the active metabolite) of the source chemicals were substituted with the values of the target chemical, methyleugenol, as were the partition coefficients, predicted using the approach of Brown et al. [18] and the chemical specific log Kow value (obtained from EPISUITE ver 1.69).
2. In addition to the above changes, the Vmax and Km, values measured in vitro, for the formation of several metabolites formed in phase I and phase II were also substituted from those relevant to estragole to those relevant for methyleugenol. These values were available for estragole, safrole and methyleugenol [14], [19], [20].

Model equations were coded and numerically integrated in Berkeley Madonna version 8.3.18. (Macey and Oster, UC Berkeley, CA, USA; https://berkeley-madonna.myshopify.com/) using the Rosenbrock’s algorithm for stiff systems. Model codes can be found in Supplementary information (appendix I).

#### 2.3.1 Alkenylbenzenes model parametrisation

In order to run the PBK models, the parameters for estragole, safrole and methyleugenol were used, as shown in Table 1 with the human physiology described in Punt et al. [14] and fixed values applied (blood flow and scaling factors) to all the PBK models as shown Table 2.

**Table 1:** Physiological parameters and scale-up factors used in the source PBK models (estragole and safrole).

Parameter name	Value	Unit
Body weight	60	Kg
Cardiac output	15	(L/hr/-1kg)
Fractional blood flow to fat - QFC	0.052	
Fractional blood flow to liver –QLC	0.227	
Fractional blood flow to richly perfused tissues QRC	0.70-QLC	
Fractional blood flow to slowly perfused tissues - QSC	0.30-QFC	
Fraction fat tissue VFC	0.214	
Fraction of liver – VLC	0.026	
Fraction richly perfused tissue - VRC	0.076-VLC	
Fraction slowly perfused tissue – VSC	0.81-VFC-VBC	
Fraction of blood [21]	VBC = 0.079	
Scaling Factors Liver		
S9PL	143	Liver S9 protein yield (mg/gram liver) [22]
MPL	32	Liver microsomal protein yield (mg/gram liver) [23]
Simulation time	24	H
Oral dose given	0.07	Mg/kg BW

**Table 2:** Kinetic parameters for phase I and phase II metabolism in liver of source and target chemicals.

	MW/ Hydroxyl metabolite MW	Vmax/Km	Partition coefficient (PC)
Estragole source [14], [15], [20], [24]	148.2/164.2 LogKow = 3.47	{Phase I} Max rate of metabolism (nmol min-1(mg protein)-1) VmaxLHEc = 0.7 ± 0.04; HE = 1′-hydroxyestragole, VmaxLAPc = 0.4 ± 0.02; AP = 4-allylphenol, VmaxLEEc = 0.9 ± 0.07; EE = estragole-2′,3′-oxide, VmaxLHAc = 1.4a ± 0.05; HA = 3′-hydroxyanethole, Vmax5 = 0.18 ± 0.02 Affinity constant (umol/L) KmLHE = 21 ± 6; KmLAP = 290 ± 28 KmLEE = 83 ± 17 KmLHA = 350 ± 20 Km5 = 618 ± 164 {Phase II} Max rate of metabolism (nmol min-1(mg protein)-1) VmaxLHEGc = 0.29; HEG = 1′-hydroxyestragoleglucuronide, VmaxLHESc = 0.006 ± 0.005; AHE = 1′-sulfooxyestragole, VmaxLOEc = 3.2 ± 0.88 Affinity constant (umol/L) KmLHEG = 708; KmLHES = 727 ± 185 KmLOE = 345 + 151	PFE = 105; Fat/blood PC; PRE = 6.5;Richly perfused tissues/blood PC PSE = 4.0;Slowly perfused tissues/blood PC PLE = 6.5;Liver/blood PC {1′-hydroxy-met} PLHE = 1.6; Liver/blood PC
Safrole [16]	162.19/ 178.18	{Phase I} metabolites of safrole, unscaled maximum rate of metabolism (nmol min-1 (mg protein)-1) VmaxLDHSc = 0.07 ± 0.004; DHS = dihydroxysafrole VmaxLHSc = 0.15 ± 0.008; HS = 1′-hydroxysafrole VmaxLHISc = 0.11 ± 0.01; HIS = 3′hydroxysafrole VmaxLCHAVc = 0.85 ± 0.05; CHAV = Dihydroxychavicol metabolites of safrole, affinity constants (umol/L) KmLDHS = 41 ± 10 KmLHS = 35 ± 10 KmLHIS = 255 ± 99 KmLCHAV = 172 ± 30 {Phase II} metabolites of 1′-hydroxysafrole, unscaled maximum rate of metabolism (nmol min-1 (mg protein)-1) VmaxLHSGc = 0.1 ± 0.006; HSG = 1′-hydroxysafrole glucuronide VmaxLHSSc = 0.017 ± 0.005; HSS = 1′H1-sulfooxysafrole VmaxLHSOc = 7.5 ± 0.4; HSO = 1 oxo safrole metabolites of 1′-hydroxysafrole, affinity constants (umol/L) KmLHSG = 1322 ± 208 KmLHSS = 3828 ± 1801 KmLHSO = 549 ± 84	PKS = 6.65; kidney/blood partition coefficient PLS = 6.65; liver/blood partition coefficient PFS = 106; fat/blood partition coefficient PRS = 6.65; richly perfused tissues/blood partition coefficient PSS = 4.2; slowly perfused tissues/blood partition coefficient; 1′-hydroxysafrole PLHS = 1.65 ;liver/blood partition coefficient
Methyleugenol target [19]	178.2/194.2 LogKow = 3.03	{Phase I} Max rate of metabolism (nmol min-1(mg protein)-1) VmaxLHEc = 1.38 ± 0.38; HE = 1′-hydroxymethyleugenol, VmaxLAPc = 0.15 ± 0.02; AP = eugenol, VmaxLEEc = 0.66 ± 0.11; EE = Methyleugenol-2′,3′-oxide, VmaxLHAc = 0.39 ± 0.08; HA = 3-(3,4-dimethoxyphenyl)-2-propen-1-ol, VmaxL5 = 0.21 ± 0.02; 3-hydroxy-4-methoxyallylbenzene, VmaxL6 = 0.10 ± 0.02; 2-hydroxy-4,5-dimethoxyallylbenzene Affinity constant (umol/L) KmLHE = 404 ± 195; KmLAP = 13.6 ± 12.3 KmLEE = 23.7 ± 5 KmLHA = 161 ± 90 KmL5 = 1097 ± 142 KmL6 = 415 ± 84 {Phase II}Max rate of metabolism (nmol min-1(mg protein)-1 VmaxLHEGc = 0.66 ± 0.087; HEG = 1′-hydroxyestragoleglucuronide,) VmaxLHESc = 0.0009 ± 0.0002; AHE = 1′-sulfooxyestragole, VmaxLOEc = 2.1 ± 1.83 affinity constant KmLHEG = 2393 ± 486 (umol/L) KmLHES = 139 ± 82 KmLOE = 1774 ± 2997	PFE = 103; Fat/blood partition coefficient PRE = 6.2; Richly perfused tissues/blood partition coefficient PSE = 3.9; Slowly perfused tissues/blood partition coefficient PLE = 6.2; Liver/blood partition coefficient {1′-hydroxy-met} PLHE = 1.4; Liver/blood partition coefficient

Sensitivity analysis was performed to identify which parameters have the greatest impact on the PBK model predictions on the formation of 1′-hydroxyestragole and 1′-sulfooxyestragole. The sensitivity analysis was performed for the source chemicals as reported by the original authors [14], [16], by using normalised sensitivity coefficients (SC) determined using the following equation:

$$\text{SC}=\left(C^{\prime }-\text{C}\right)\;/\;\left(P^{\prime }-\text{P}\right)*\left(\text{P}/\text{C}\right)$$

where C is the initial value of the model output; C′ is the modified model output resulting from a 5% increase in the parameter value; P is the initial parameter value; and P′ is the modified parameter value [25]. A 5% increase in parameter values was chosen to analyze the effect of a change in parameter values on formation of 1′-hydroxyestragole and 1′-sulfooxyestragole at a dose 0.07, mg/kg bw/day for 24 h exposure, representing a realistic daily intake [26]). Each parameter was analysed individually, while other parameters were kept as their initial value. Sensitivity analysis was conducted on the estragole PBK model, to identify the most sensitive parameters, which were identified as the Vmax and Km kinetic constants (results not shown).

#### 2.3.2 Alkenylbenzenes analysis using derived PBK models

In order to provide evidence of this proof-of-concept, the analysis was carried out in three steps.

1. Using only the original model of estragole (ES), safrole (SA) and methyleugenol (ME). The results were named with the following label ES_original, SA_original and ME_original;
2. In the second step, only the molecular weight (MW) and partition coefficient (PC) was changed to that of the target chemical (ME); the model and results are referred to as ES_ME_MW_PC or SA_ME_MW_PC.
3. The third step of the analysis was to change also the biochemical (biotransformation) parameters to those of the target chemical, such as, Vmax and Km of each metabolite. The model code and results were named ES_ME_all and SA_ME_all.

The PBK model codes of the source chemicals are reported in appendix I (supplementary information) along with the original PBK model code for methyleugenol (based on Al-Subeihi et al. [19]), used in the present proof-of-concept evaluation of the model.

**Fig. 3:**
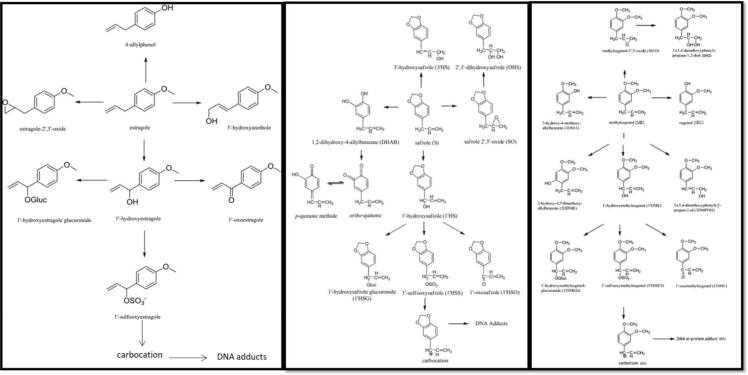
Proposed metabolic pathways of the alkenylbenzenes: estragole (right [14]), safrole (middle [16]) and methyleugenol (left [19]).

The PBK model predictions for methyleugenol were based on the proposed biotransformation pathways of the source chemicals. The difference in metabolism between estragole, safrole and methyleugenol is that methyleugenol has an additional two metabolites formed, 3,4 dimethoxyphenyl)-2-propen-1-ol (3DMPOH) and 2-hydroxy-4,5-dimethoxyallylbenzene (2HDME), that do not appear in estragole and safrole pathways so far published in the literature [14], [16], [20] probably due to the instrument analytical sensitivity. Recently, an additional metabolite formed in phase I for estragole was quantified and introduced in a newly revised version of the estragole PBK model [15]. This last PBK model code was used for the final analysis since it was available as supplementary information [15]. However, the main pathway leading to the adverse outcome is via DNA adduct binding by hydroxylation, and is common for the three chemicals. Using the human PBK models for estragole, a comparison could be made between the model predictions in formation of the 1′-hydroxy metabolite (Fig. 3).

## 3 Results

### 3.1 Identification of potential analogues

Step 1 of the workflow shown in Fig. 1 is the identification of potential analogues. Literature searching, using a range of electronic resources, was undertaken to identify potential analogues of methyleugenol, belonging to the family of alkenylbenzenes, for which suitable PBK models were available. This included searching for analogues within the OECD QSAR Toolbox (ver 4.4) and in the Knowledgebase of Lu et al. [6] that had been enriched with additional models found in the literature. An in-house KNIME workflow was used to identify chemicals from within the enriched Lu et al Knowledgebase [6] that were ranked most frequently in the top five most similar chemicals. For chemicals that appeared most frequently in the top five most similar chemicals (i.e. identified by at least four similarity metrics), an average similarity value was calculated. Table 3 shows the potential analogues that were identified, along with additional information regarding where these were obtained from, similarity scores and the availability of PBK models associated with the chemicals (more information on this analysis is reported in the excel document as Supplementary information – appendix II).

**Table 3:** Potential analogues of methyleugenol, similarity scores and availability of human or rodent PBK models.

Chemical	Tanimoto similarity score (OECD QSAR Toolbox)	Average similarity score (KNIME workflow)	Reference for human PBK model	Reference for rodent PBK model	Validity / reproducibility of PBK model
Eugenol	0.82	(excluded as full model unavailable)	[27]	–	Irreproducible - full model not reported.
Elemicin	0.80	0.78	[28] PBK model developed using read across approach	[28] PBK model developed using read across approach	Not validated with in vivo data
Estragole	0.70	0.66	[14], [15], [20]	[29], [30], [31]	Valid and reproducible
Safrole	0.64	0.69	[16]	[16]	Valid and reproducible
Myristicin	0.66	0.58	[32] PBK model developed using read across approach	[32] PBK model developed using read across approach	Not validated with in vivo data
Apiol	< 0.6	0.49	[33]	[33]	Not validated with in vivo data
Isosafrole	< 0.6		Not available	Not available	–
Anethole	< 0.6		Not available	Not available	–
Ally benzene	< 0.6		Not available	Not available	–
Methyleugenol*	1.00	1.00	[19]	[34]	Valid and reproducible*

* Reference to methyleugenol is for completeness; this model was used to validate the approach, demonstrate the proof-of principle after characterisation and parameterisation of the model derived from analogues.

Characterisation of the target chemical and analogues was performed by using a matrix similar to the ones used within the OECD IATA Case Studies project (see excel document in Supplementary material – appendix II). The information was retrieved from available e-resources and tools such as OECD QSAR Toolbox.

### 3.2 Selection and justification of analogue choice

Step 2 of the workflow shown in Fig. 1 is the selection and justification of analogue choice from the list of potential analogues obtained in step 1. The two following criteria were used to determine the most appropriate analogues for the present work, according to the strategic flowchart depicted in Fig. 2.

1. Similar ADME processes and known, relevant mode of toxic action
2. Availability of an existing PBK model that has been validated against in vivo data

Table 3 shows that five chemicals namely, eugenol, elemicin, estragole, safrole and myristicin have been identified as potential analogues based on structural similarity (all others having a similarity score < 0.6). The structural similarity scores are based on presence of common structural features. On the other hand, mechanistic similarity is supported by potential formation of a common 1′-hydroxy metabolite for all of these chemicals as predicted by the mammalian metabolism simulators in the OECD QSAR Toolbox (ver 4.4). The PBK model simulations for eugenol were predicted using Gastroplus [27]; however, insufficient information regarding the model code were available to enable it to be reproduced and no evaluation was performed against in vivo data, hence this chemical was excluded. The PBK models for myristicin and elemicin were parametrised using in vitro measured data and evaluated by comparing to other alkenylbenzene in a read across approach [28], [32] and were therefore also excluded since they did not have specific evaluation against in vivo data for the specific chemical. Thus, after applying the above selection criteria, the shortlisted potential analogues were estragole and safrole. Therefore, in this analysis both were used to increase the weight of evidence of the approach.

### 3.3 Use of analogues in PBK modelling

Step 3 of the workflow uses the selected analogues to derive a PBK model for the target chemical. Both safrole and estragole had an available human PBK model, evaluated against literature data. The PBK models were run to check that the mass balance was stable. The mass balance did not report any error reflecting that the models were properly built. Fig. 3 demonstrates the known similarity in metabolism for estragole, safrole and methyleugenol (which is pertinent to the proposed mechanism of toxic action), hence the PBK model code of these two chemicals were used to run simulations for methyleugenol. The full PBK model description and evaluation can be found in the supplementary material of the OECD PBK model guidance document [4]. Description of the model and development of the case study was performed following the required criteria [35], recorded using the established template and evaluated using the available checklist [4].

The PBK model developed here for methyleugenol was based on the proposed biotransformation pathways of estragole and safrole. Fig. 3 shows the different pathways for the chemicals highlighting the difference in metabolism between estragole, safrole and methyleugenol. Methyleugenol has an additional two metabolites formed in phase I (3,4 dimethoxyphenyl)-2-propen-1-ol (3DMPOH) and 2-hydroxy-4,5-dimethoxyallylbenzene (2HDME)), that do not appear in the safrole and estragole pathway so far published in the literature. Recently a fifth metabolite for estragole was identified and measured by Ning et al. [15] and introduced in the estragole PBK model. On the other hand the safrole PBK model was complete with additional metabolism also occurring in the kidney, which was not the case in the estragole model. However, the main pathway leading to the adverse outcome, DNA adduct binding, is via hydroxylation; this pathway is common for all alkenylbenzene chemicals due to their genotoxic action.

Fig. 4 reports the predictions of the amount of the chemical that reaches the liver (at 24hrs using an external dose of 0.07 mg/kg BW). The predictions were run for estragole (Fig. 4A-C) and safrole (Figure Fig. 4D-F) and using chemical information for the target methyleugenol. The results show that the peak of methyleugenol in the liver, when input values were altered to those relevant for methyleugenol, gave similar Cmax values - around 1 µmol. While when changing only the MW and PC, the amount in liver predicted by the safrole model was 2 µmol compared to the amount predicted by the estragole model which was 1.2 µmol. All the values are very close but using the safrole model a slight overestimation could occur. In order to see which model was closest, a model for methyleugenol was built using the information reported in Al-Subeihi et al. [19]; results are shown in Fig. 4G). The predictions using the PBK model from the selected source chemicals were very close to the original ME model. Evaluation of the prediction compared to in vivo data was performed according to the method of Al-Subeihi et al. [19] using methyleugenol concentrations in serum blood of human volunteers at different time points following intake of methyleugenol from cookies [36]). A dietary dose of 0.00075 mg methyleugenol/kg bw was used for simulations with a timeframe of two hours to mimic the in vivo study; results are shown in Fig. 4H.

**Fig. 4:**
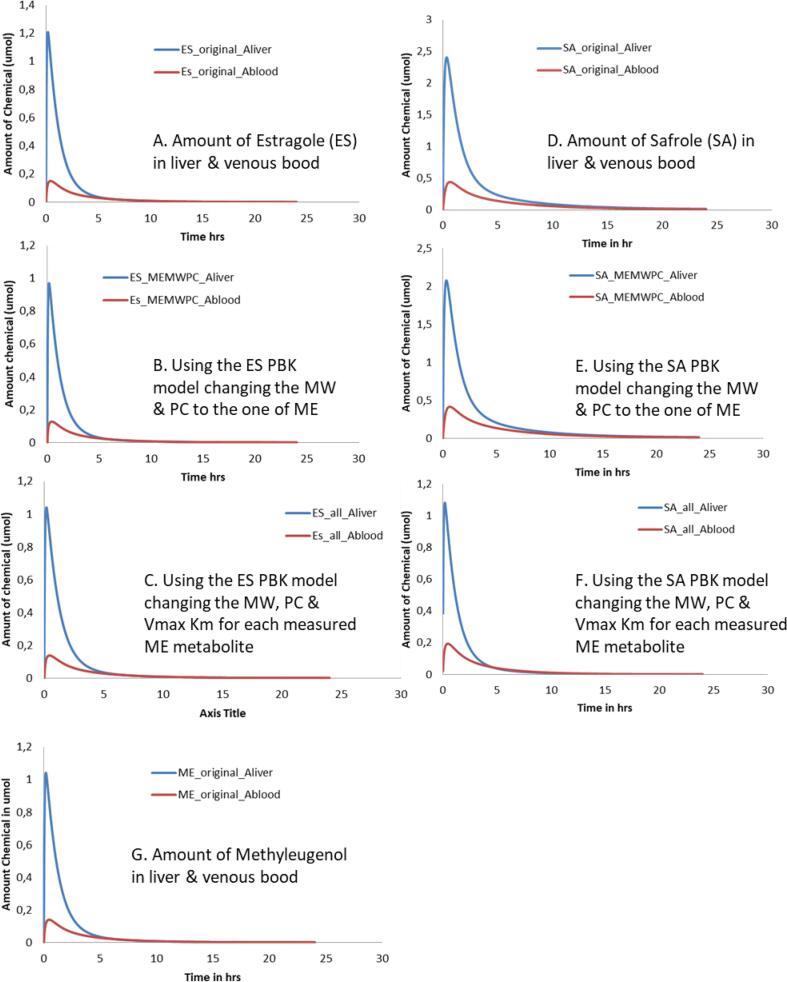
PBK model predictions of the amount of the chemical reaching the liver and venous blood (at 24hrs using a dose of 0.07 mg/kg BW) for estragole (4A-C) and safrole (4D-F) and when the parameters are changed to those for the target chemical – methyleugenol. Fig. 4G PBK model predictions of the amount of methyleugenol reaching the liver (at 24hrs using a dose of 0.07 mg/kg BW) using the original PBK model described in Al-Subeihi et al. [19].

**Fig. 4H:**
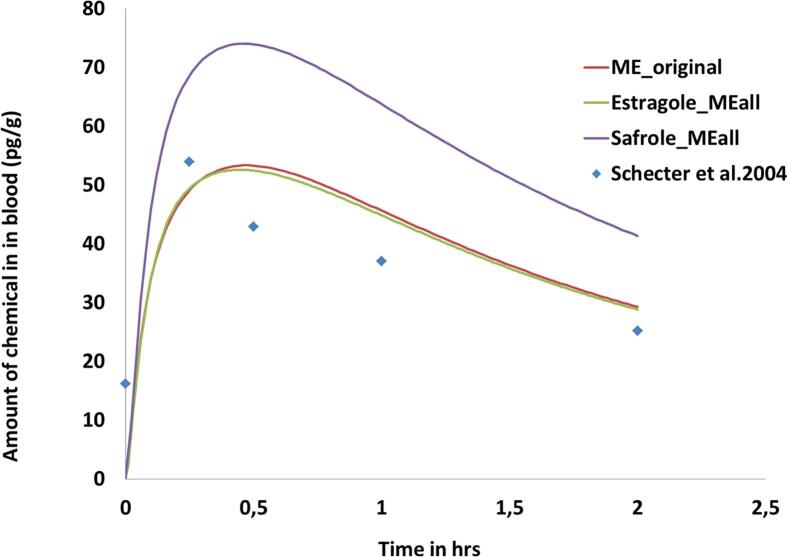
Simulation of the amount of methyleugenol in blood, predicted using the source PBK models (safrole and estragole) adapted to use input data relevant to methyleugenol versus the original PBK model for methyleugenol and the in vivo data from Schecter et al. [36]. The dose was adapted to a dietary intake of 0.00075 mg/Kg bw.

**Fig. 5:**
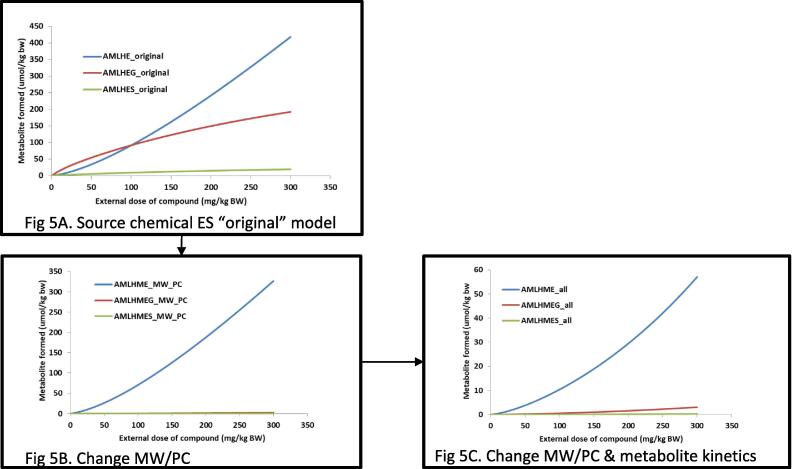
External dose response of chemical versus the formation of the hydroxyl (AMLHME), sulfo (AMLSHE) and glucuronide (AMLGME) metabolites in liver. AMLH = amount in liver of the metabolite; ME = methyleugenol. Each line represents simulation of the increasing external dose of the parent compound (0 – 300 mg/kg BW) versus the concentration of the internally formed metabolite, hydroxyl, glucuronidation, sulfation. Fig. 5A is the predictions based on the original PBK model for estragole; Fig. 5B was achieved using the estragole model but changing the MW and partition coefficient values to those of methyleugenol and its metabolite; Fig. 5C, changing also the in vitro kinetic constant in the model to the one of methyleugenol metabolite, but still based on the proposed biotransformation pathways of estragole.

**Fig. 6:**
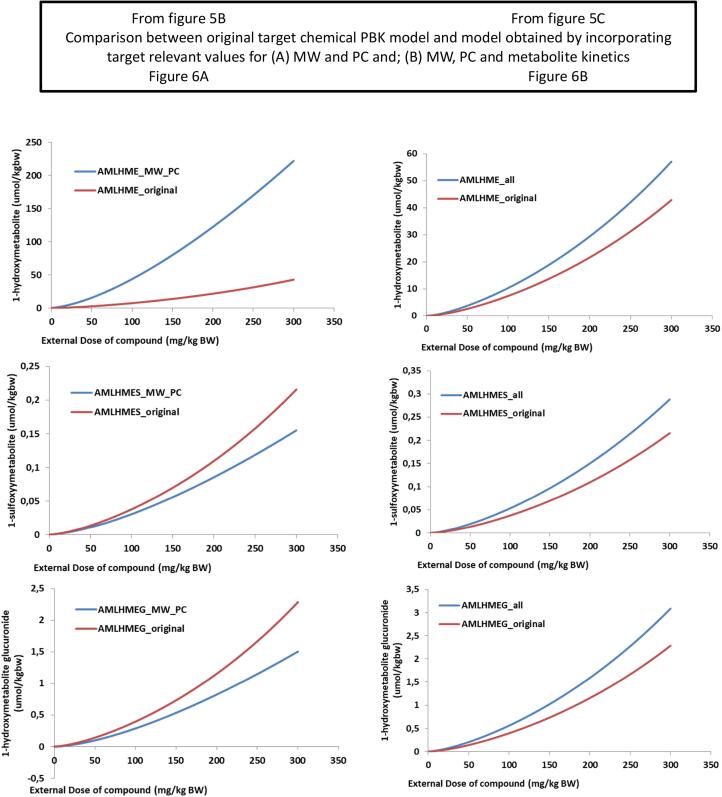
Taking Fig. 5A and C which now represents Fig. 6A and 6B respectively. Comparison of the external dose-response formation of three metabolites formed, (1) hydroxylation (AMLHME), (2) sulfation (AMLSME), (3) glucuronidation (AMLGME), using the estragole model in a read across manner versus the original methyleugenol (ME). Part A = estragole model but changing the MW and PC values of methyleugenol and its metabolites; Part B changing also the kinetic constant of methyleugenol metabolite based on the proposed biotransformation pathways of estragole.

Fig. 5 depicts the results of the simulation carried out using the estragole PBK model but changing the chemical specific input information, MW and partition coefficient, to the one of the target chemical. The results show the external dose response of chemical versus the formation of the hydroxyl (AML**H**ME), sulfoxy (AML**S**ME) and glucuronide (AML**G**ME) metabolites (where AMLH is the amount in liver of the metabolite and ME = methyleugenol). Each line represents simulation of the increasing external dose in mg/kg BW of the parent compound versus the concentration of the internally formed metabolites, hydroxyl, glucuronide and sulfate. At the top (Fig. 5A) are the simulations based on the original PBK model for estragole, without any changes in the input parameters; in the middle (Fig. 5B) are the simulations using the estragole model but changing the MW and partition coefficient values to those of methyleugenol and its metabolite; Fig. 5C reports simulations when also the in vitro kinetic constant for metabolite formation are changed in the model to the one of methyleugenol metabolite, but still based on the proposed biotransformation pathways of estragole (values available in Table 2). Finally, the predictions obtained for the three main metabolites formed (hydroxyl, sulfoxy and glucononidation) were compared to the original methyleugenol (ME) model predictions using the ME code described in Al-Subeihi 2011 [34] (Fig. 6). All simulations were run with a 24hrs window and an external dose given to the model of 0.07 mg/kg bw of the chemical. The PBK model predictions for each chemical and specific code can be identified by the following label ES-ME-SA_original (predictions using the original code); by changing the Molecular Weight (MW) and partition coefficient (PC) the label is ES-SA_MW_PC (to the original code of ES or SA the values of ME were inputted) and by changing Vmax and Km it is labelled ES-SA_all (in addition to the MW, PC also the Vmax and Km for ME were introduced).

**Fig. 7:**
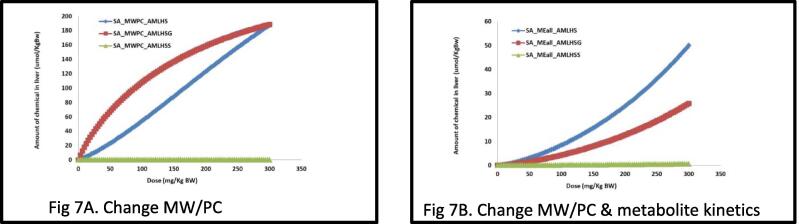
External dose response of chemical versus the formation of the hydroxyl (AMLHME), sulfo (AMLSHE) and glucuronide (AMLGME) metabolites in liver. AMLH = amount in liver of the metabolite; ME = methyleugenol. SA = Safrole. Each line represents simulation of the increasing external dose of the parent compound (0 – 300 mg/kg BW) versus the concentration of the internally formed metabolite, hydroxyl, glucuronidation, sulfation. Fig. 7A was achieved using the safrole model but changing the MW and partition coefficient values to those of methyleugenol and its metabolite; Fig. 7B, changing also the in vitro kinetic constant in the model to the one of methyleugenol metabolite, but still based on the proposed biotransformation pathways of safrole.

**Fig. 8:**
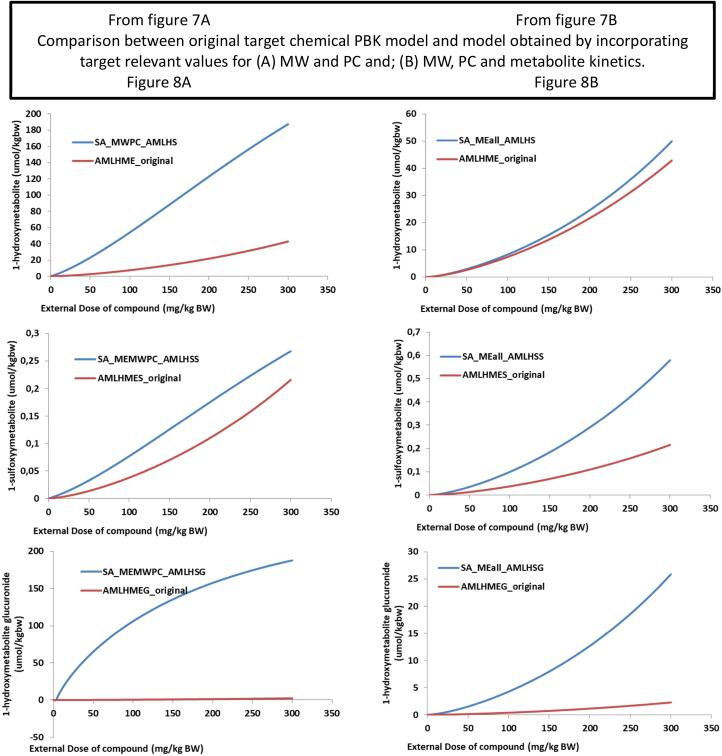
Taking Fig. 7A and B which now represents Fig. 8A and B respectively. Comparison of the external dose-response formation of three metabolites formed, (1) hydroxylation (AMLHME), (2) sulfation (AMLSME), (3) glucuronidation (AMLGME), using the safrole model in a read across manner versus the original methyleugenol (ME). Part A = safrole model but changing the MW and PC values of methyleugenol and its metabolites; Part B changing also the kinetic constant of methyleugenol metabolite based on the proposed biotransformation pathways of safrole.

The same analysis was carried out using the safrole model; when changing only the MW and PC, this resulted in a higher amount of the glucuronide metabolite to be formed (Fig. 7A and 8A). When changing also the biokinetic information (Fig. 7B, 8C) this resulted in a similar representation as predicted with the estragole model (Fig. 4, Fig. 5).

### 3.4 Reporting

The final step of the workflow shown in Fig. 1 is adequate reporting of the model. The OECD PBK model reporting template and evaluation checklist were applied to report and assess the PBK model for methyleugenol using the model code from estragole [4]. Model validity was established using the principles outlined in the OECD guidance document [35], [4])

## 4 Discussion

The present study provides a strategy to identify suitable “data-rich” analogues (source chemicals) for a “data-poor” chemical of interest (target chemical) for which there is a need to develop and apply a new PBK model. The study also provides an example of PBK model evaluation, using in vivo biokinetic data from (structural) analogues of a chemical of interest. A strategic flowchart is described that complements the workflow presented in the OECD guidance document [4]. The four steps in the workflow are: *Identification of potential analogues; selection and justification of analogues; use of analogues in PBK modelling; and reporting.*

The purpose of this study was to provide a means to develop and evaluate a PBK model for a chemical for which no in vivo reference data were available. Existing information, not only on ADME and TK parameters, but also on effect data such as toxicity data (in the present case - formation of DNA adducts, as a biomarker of effect), from the analogues were also used to make a prediction for the target. The present workflow was applied using methyleugenol as the target chemical. Methyleugenol is an alkenylbenzene that occurs naturally in various herbs such as tarragon, basil, and nutmeg and occurs at low levels in oranges, bananas, and grapefruit juices [37]. It is of interest as it is known to be genotoxic at high doses in rodent studies. The safety of human exposure to methyleugenol at low dietary intake levels is relevant under the food safety law. It should be noted that this was a hypothetical scenario as a PBK model is available for methyleugenol; this was used to validate the proof-of-principle.

### 4.1 Identification of potential analogues

The principle of read-across (i.e. using data rich (source) analogues to make inferences regarding similar chemicals that are data poor (targets) is well-established. Success of the approach (for example, regulatory acceptance) is dependent on the strength of the supporting arguments, rationalisation of analogues and provision of adequate information, as discussed by Ball et al. [38]). Read-across for the purposes of assisting safety assessment is strengthened by incorporating toxicokinetic information into the justification of analogue selection. Laroche et al., reporting the results from a European Partnership for Alternative Approaches to Animal Testing (EPAA) Partners' Forum, provide several examples where toxicokinetic information has been used, across a range of industries, to support read-across predictions [39]. The report also identified several areas where more research is needed; one area identified is the need to establish appropriate similarity metrics for identifying analogues. There is no absolute, universally accepted, criteria by which the “similarity” of one chemical to another can be unequivocally determined. This means that in selecting appropriate analogues to use as source chemicals there are several criteria to consider. The criteria by which one chemical may be considered similar to another includes similarity in physicochemical properties, chemical structure, ADME processes, metabolite formation, mode of toxic action etc. One constraint, in terms of selecting a suitable analogue for read-across, is ensuring that the analogue has sufficient, relevant data associated with it. This is a particularly limiting constraint in terms of finding analogues for which a PBK model is available, as there are relatively few - in comparison to the number of chemicals for which safety assessment is required. Lu et al. proposed the use of a PBK model from a data rich chemical to inform development of a new PBK model for a data poor chemical [6]. In their analysis, similarity was determined by the construction of a correlation matrix between target and source chemicals, relating to eight molecular properties associated with pharmacokinetic behaviour: MW, hydrogen bond acceptor count, hydrogen bond donor count, number of rotatable bonds, polar surface area, partition coefficient, solubility and surface area. The work of Ellison et al. [7], [8] demonstrated that both structural and functional TK analogues were suitable for providing TK information that could be used to evaluate a PBK model for a data poor chemical. Structural analogues were identified by considering similarity in chemical scaffold, functional groups, metabolism and physico-chemical properties and similarity score; functional analogues were identified by considering Biopharmaceutics Drug Disposition Classification System (BDDCS) class, Extended Clearance Classification System (ECCS) class, potential for being a p-glycoprotein substrate, oral bioavailability, volume of distribution and systemic clearance [7].

In this analysis potential analogues and relevant PBK models were sought using extensive literature searching, investigation of the OECD QSAR Toolbox (ver 4.4) and identification of similar chemicals from an enriched version of the Lu et al. Knowledgebase, using an in-house KNIME workflow. A small number of potential candidates were identified at this stage.

### 4.2 Selection and justification of analogues

In the scenario described in this paper, analogue selection is significantly constrained, as only chemicals for which a valid PBK model, evaluated against in vivo data, exists can be considered. As outlined in Section 3.2, potential analogues are identified on the basis of structural similarity (here determined using a combination of similarity metrics) and then must meet two additional criteria in order to be selected. These are: similarity in ADME processes and mechanism of toxic action (criterion 1); and the availability of a valid and reproducible human PBK model evaluated with in vivo data (criterion 2). This demonstrates that structural as well as mechanistic similarity formed the key criteria for the selection of analogues. The structural similarity was based on presence of common structural features; mechanistic similarity was based on metabolic pathways. The mammalian metabolism simulators present in the OECD QSAR Toolbox (ver 4.4) predicted formation of a common 1′-hydroxy metabolite for each of the candidate analogues. This mechanism is characteristic of this class of compounds and it involves initial hydroxylation of the benzylic carbon of the alkenyl side chain catalyzed by cytochrome P450 followed by formation of an electrophilic carbocation that binds to DNA [40]. Application of both structural and mechanistic similarity criteria resulted in two potential analogues: estragole, and safrole. Estragole and safrole were selected with the highest structural similarity score (0.70 and 0.68), and with available valid human PBK models [14], [16] with similarity in ADME processes to the target chemical, methyleugenol. The model structure reflected the WHO principle of ensuring that models are as simple as practicable [41] but takes into account metabolism formation as the main pathway to the adverse effect; thus underlining the toxicological relevance (leads to DNA adduct formation) of the model structure and parameters. The internal consistency (robustness) was achieved by reporting what is plausible, reflecting the mechanistic understanding of biology and biochemistry; the mass balance of the model reported no error and the results were consistent.

### 4.3 Use of analogues in PBK modelling

Using the valid human PBK models for estragole and safrole [14], [15], [16], the input parameters were changed to those of methyleugenol, simulations were carried out for the main phase I metabolite (1′-hydroxy) and the two main metabolites formed in phase II (sulfation and glucuronidation). Fig. 5A reports simulations (at the same external conditions, time and dose) of these three metabolites for the source chemical (estragole). Fig. 5B and C depict the source chemical model when the input parameters are changed to those of the target chemical. This step (of changing the input parameters) was done in two stages, to show the difference obtained when a minimal data set (of only Log Kow –partition coefficients- and MW) is available and when additional input values (Vmax and Km representing metabolites formed) are available. The simulations of metabolites formed, showed a 2-fold difference (for the two phase II metabolites) between the two scenarios (Fig. 5B and C) however, for the 1-hydroxymetabolite there was 4-fold difference. Comparing the two simulations (Fig. 5B and C) shows that metabolism information of the target chemical plays a key role in the fate of the chemical in the body and can influence its kinetics and dynamics. It is therefore recommended, wherever possible, to include information relating to the metabolism. However, if information on metabolism is not available, then (as in the present case) incorporating information only relating to the MW and Log Kow provides a reasonable result.

To demonstrate the proof-of-concept for the principle underlying this work (i.e. that a valid PBK model from an analogue (source) chemical could be used as a template for a target chemical) the original PBK model built for methyleugenol [19]) was used and the simulation results obtained by the two models were compared. In addition the predictions were evaluated using the in vivo data for methyleugenol as reported [19]. All the simulations (Fig. 5A and B) showed overestimation of the metabolite formation using the estragole model with the methyleugenol input parameters. The overestimation was lower or slightly above the 2-fold considered reasonable by WHO [41]. However, when changing the MW and PC values to those of methyleugenol, the prediction of the phase II metabolites, sulfation and glucuronidation, were underestimated as compared to the original PBK model code for methyleugenol. When changing in the PBK model code of estragole all the input values, MW, PC, and Vmax and Km, the 1′-hydroxy metabolite simulation was consistent between the methyleugenol model developed using the estragole code as a template and the original model. This shows that the current approach of using a valid PBK model from a source chemical to provide information to develop a PBK model for a target chemical, of sufficient similarity, can be carried out. The read-across approach showed external consistency, demonstrating a consistent quantitative relationship of the model simulations achieved using the estragole model versus the original methyleugenol model. In addition the approach could be used to identify and understand, qualitatively, the biokinetic processes of a data poor chemical.

### 4.4 Reporting

Detailed and accurate reporting of PBK models is essential for their uptake and acceptance, particularly in the regulatory context, as the models need to be transparent and reproducible. Here, we have completed the PBK model reporting format as proposed in the OECD guidance document [4]. The detailed report is available as Supplementary information (Appendix III).

## 5 Conclusions

In conclusion, the validity of the estragole and safrole human PBK models has already been established [14], [16]; herein their applicability for the kinetic modelling of methyleugenol has been demonstrated. On the basis of the results obtained from this study, it was concluded that using kinetic data from a source chemical (estragole or safrole) to make a read across argument for a target chemical (methyleugenol) is a reasonable approach to inform a safety assessment. This uses all information available, on hazard and toxicokinetics, in the absence of in vivo data to validate the methyleugenol PBK model. Furthermore, the application of a PBK model that takes into account the biokinetics and biotransformation of the chemical of interest reduces the uncertainties in the absorption, distribution, metabolism and excretion characteristics of the chemical. This approach can be thought of as a “read-across” approach to rapidly use a valid PBK model to obtain predictions that could support and provide mechanistic insight for the assessment of a data-poor chemical.

## Declaration of Competing Interest

The authors declare that they have no known competing financial interests or personal relationships that could have appeared to influence the work reported in this paper.
