# Supplementary material for: Assessment of the predictive capacity of a physiologically based kinetic model using a read-across approach
Source: Comput Toxicol. 2021 May;18:100159. doi: 10.1016/j.comtox.2021.100159 (PMC8130669; doi:10.1016/j.comtox.2021.100159)
Supplement: Supplementary data 1 [file mmc1.docx]

**Annex 1** PBK model codes

; Data: 02-05-2011 based on human model of 2009

; Purpose: PBK Final Human Model Estragole

; Species: Human

; Compiled by: Ans Punt & Alicia Paini

; Organisation: Wageningen University

;================================================================================

;Physiological parameters

;================================================================================

;Tissue volumes

BW = 60 {Kg} ; body weight human

VLc = 0.026 ; fraction of liver tissue

VKc = 0.004 ; fraction of kidney tissue

VLuc = 0.008 ; fraction of lung tissue

VFc = 0.214 ; fraction of fat tissue

VAc = 0.02 ; fraction of arterial blood: 0.079*1/4

VVc = 0.059 ; fraction of venous blood: 0.079*3/4

VRc = 0.08-VLc-VLuc-VKc ; fraction of richly perfused tissue

VSc = 0.836-VFc-VAc-VVc ; Fraction of blood flow to slowly perfused tissue

; total of fractions = 0.916

VL = VLc*BW {L or Kg}

VLu= VLuc*BW

VK = VKc*BW

VF = VFc*BW

VR = VRc*BW

VS = VSc*BW

VA = VAc*BW

VV = VVc*BW

;------------------------------------------------------------------------------------------------------------------------------------------------------------------------------------

;Blood flow rates

QCc = 15 ; Info: QC=15*BW^0.74 Reference: Brown

QC = QCc*BW**0.74 {L/hr} ; Info: QC=15*BW^0.74 Reference: Brown

QLc = 0.227 ; Fraction of blood flow to liver

QKc = 0.175 ; Fraction of blood flow to kidney

QFc = 0.052 ; Fraction of blood flow to fat

QRc = 0.70-QLC-QKC ; Fraction of blood flow to richly perfused tissue

QSc = 0.30-QFC ; Fraction of blood flow to slowly perfused tissue

; total of fractions = 1

QL = QLc*QC {L/hr}

QK = QKc*QC {L/hr}

QF = QFc*QC {L/hr}

QR = QRc*QC {L/hr}

QS = QSc*QC {L/hr}

;================================================================================

;Partition Coefficients

;================================================================================

;estragole (E)

PLE = 6.5 ; liver/blood partition coefficient

PLuE=6.5 ; lung/blood partition coefficient

PKE=6.5 ; kidney/blood partition coefficient

PFE = 105 ; fat/blood partition coefficient

PRE = 6.5 ; richly perfused tissues/blood partition coefficient

PSE = 4.1 ; slowly perfused tissues/blood partition coefficient

;1'-hydroxyestragole (HE)

PLHE = 1.6 ;liver/blood partition coefficient

;================================================================================

;Biochemical parameters

;================================================================================

;Linear uptake rate (hr-1)

Ka = 1

;------------------------------------------------------------------------------------------------------------------------------------------------------------------------------------

;Metabolism liver

;Scaling factors

S9PL=143 ; Liver S9 protein yield (mg/gram liver) Pang et al.1985

MPL=32 ; Liver microsomal protein yield (mg/gram liver) Atio et al. 1976

L=VLC*1000 ; Liver = 26 (gram/kg BW)

;metabolites of estragole, unscaled maximum rate of metabolism (nmol min-1 (mg protein)-1)

VmaxLHEc = 0.73 ;HE = 1'-hydroxyestragole,

VmaxLAPc = 0.38 ;AP = 4-allylphenol

VmaxLEEc = 0.85 ;EE = estragole-2',3'-oxide

VmaxLHAc = 1.35; ;HA = 3'-hydroxyanethole

VmaxLM5c = 0.18 ;M5 = metabolites 5

;metabolites of estragole, scaled maximum rate of metabolism (umol hr-1)

VMaxLHE = VMaxLHEc/1000*60*MPL*L*BW

VMaxLAP = VMaxLAPc/1000*60*MPL*L*BW

VMaxLEE = VMaxLEEc/1000*60*MPL*L*BW

VMaxLHA = VMaxLHAc/1000*60*MPL*L*BW

VMaxLM5 = VMaxLM5c/1000*60*MPL*L*BW

;metabolites of estragole, affinity constants (umol/L)

KmLHE = 21

KmLAP = 290

KmLEE = 83

KmLHA = 350

KmLM5 = 618

;metabolites of 1'-hydroxyestragole, unscaled maximum rate of metabolism (nmol min-1 (mg protein)-1)

VmaxLHEGc = 0.3 ; HEG = 1'-hydroxyestragole glucuronide

VmaxLOEc = 4.9 ; OE= 1'-oxoestragole

VmaxLHESc = 0.007 ; HES = 1'-sulfooxyestragole

;metabolites of 1'-hydroxyestragole, scaled maximum rate of metabolism (umol hr-1)

VMaxLHEG = VmaxLHEGc/1000*60*MPL*L*BW

VMaxLOE = VmaxLOEc/1000*60*S9PL*L*BW

VMaxLHES = VmaxLHESc/1000*60*S9PL*L*BW

;metabolites of 1'-hydroxyestragole, affinity constants (umol/L)

KmLHEG = 708

KmLOE = 354

KmLHES = 727

;================================================================================

;Run settings

;================================================================================

;Molecular weight

MWE = 148.2; Molecular weight estragole

MWHE= 164.2; Molecular weight 1'-hydroxyestragole

;Given dose (mg/ kg bw) and oral dose umol/ kg bw}

GDOSE = 0.07 {mg/ kg bw} ; GDOSE = given dose

ODOSE = (GDOSE*1E-3)/MWE*1E6 {umol/ kg bw} ; ODOSE = given dose recalculated to umol/kg bw

DOSE=ODOSE*BW; ; DOSE = umol

;Time

Starttime = 0; in hrs

Stoptime = 24; in hrs

;================================================================================;Dynamics

;================================================================================

;slowly perfused tissue compartment

;AS = Amount estragole in slowly perfused tissue, umol

AS' = QS*(CA-CVS)

Init AS = 0

CS = AS/VS

CVS = CS/PSE

;--------------------------------------------------------------------------------------------------------------------------------------------

;richly perfused tissue compartment

;AR = Amount estragole in richly perfused tissue, umol

AR' = QR*(CA-CVR)

Init AR = 0

CR = AR/VR

CVR = CR/PRE

;--------------------------------------------------------------------------------------------------------------------------------------------

;fat compartment

;AF = Amount estragole in fat tissue, umol

AF' = QF*(CA-CVF)

Init AF = 0

CF = AF/VF

CVF = CF/PFE

;--------------------------------------------------------------------------------------------------------------------------------------------

;uptake estragole from GI tract

;AGI = Amount estragole remaining in GI tract (umol)

AGI' =-Ka*AGI

Init AGI = DOSE

;--------------------------------------------------------------------------------------------------------------------------------------------

;liver compartment

;estragole

;AL = Amount Estragole in liver tissue, umol

AL' = QL*(CA -CVL)+ Ka*AGI - AMLHE' - AMLAP' - AMLEE' -AMLHA'

Init AL = 0

CL = AL/VL

CVL = CL/PLE

AUCL' = CL

init AUCL = 0

;AMLHE=Amount estragole metabolized in liver to 1'-hydroxyestragole (HE)

AMLHE' = VmaxLHE*CVL/(KmLHE + CVL)

init AMLHE = 0

CMLHE =AMLHE/VL

CMBWHE =AMLHE/BW*1000 ;nmol/kg bw

;AMLAP=Amount estragole metabolized in liver to 4-allylphenol (AP)

AMLAP' = VmaxLAP*CVL/(KmLAP + CVL)

init AMLAP = 0

CMLAP =AMLAP/VL

CMBWAP =AMLAP/BW*1000 ;nmol/ kg bw

;AMLEE=Amount estragole metabolized in liver to estragole-2',3'-oxide (EE)

AMLEE' = VmaxLEE*CVL/(KmLEE + CVL)

init AMLEE = 0

CMLEE =AMLEE/VL

CMBWEE =AMLEE/BW*1000 ;nmol/kg bw

;AMLHA=Amount estragole metabolized in liver to 3'-hydroxyanethole (HA)

AMLHA' = VmaxLHA*CVL/(KmLHA + CVL)

init AMLHA = 0

CMLHA =AMLHA/VL

CMBWHA =AMLHA/BW*1000

;AMLM5=Amount estragole metabolized in liver to M5 (M5)

AMLM5' = VmaxLM5*CVL/(KmLM5 + CVL)

Init AMLM5 = 0

CMLM5 =AMLM5/VL

;1'-hydroxyestragole

;ALHE = amount 1'-hydroxyestragole in liver tissue, umol

ALHE' = AMLHE' - AMLHEG' - AMLHES' - AMLOE'

Init ALHE = 0

CLHE = ALHE/VL

CVLHE = CLHE/PLHE

AUCLHE' = CLHE

init AUCLHE = 0

;AMLHEG= amount 1'-hydroxyestragole metabolized in liver to 1'-hydroxyestragole glucurondie (HEG)

AMLHEG' = VmaxLHEG*CVLHE/(KmLHEG + CVLHE)

init AMLHEG = 0

CLHEG = AMLHEG/VL ;nmol/g liver (umol/kg liver)

CBWHEG = AMLHEG/BW*1000 ;nmol/kg bw

;AMLOE= amount 1'-hydroxyestragole metabolized in liver to 1'-oxoestragole (OE)

AMLOE' = VmaxLOE*CVLHE/(KmLOE + CVLHE)

init AMLOE = 0

CLOE = AMLOE/VL

CBWOE = AMLOE/BW*1000

;AMLHES= amount 1'-hydroxyestragole metabolized in liver to 1'-sulfooxyestragole (HES)

AMLHES' = VmaxLHES*CVLHE/(KmLHES + CVLHE)

init AMLHES = 0

CLHES = AMLHES/VL

CBWHES = (AMLHES/BW)*1000 ;nmol/kg bw

;--------------------------------------------------------------------------------------------------------------------------------------------

;kidney compartment

;AK = amount estragole in liver tissue, umol

AK' = QK*(CA -CVK)

Init AK = 0

CK = AK/VK

CVK = CK/PKE

;--------------------------------------------------------------------------------------------------------------------------------------------

;lung compartment

;ALu = amount estragole in lung tissue, umol

ALu' = QC*(CV-CALu)

Init ALu = 0

CLu = ALu/VLu

CALu = CLu/PLuE

;--------------------------------------------------------------------------------------------------------------------------------------------

; arterial blood compartment

;CA = Concentration arterial blood estragole

AA' = QC*(CALu-CA);

Init AA = 0

CA = AA/VA

;--------------------------------------------------------------------------------------------------------------------------------------------; venous blood compartment

;CV = Concentration venous blood estragole (umol/L)

AV' = (QF*CVF + QR*CVR + QS*CVS + QL*CVL + QK*CVK - QC*CV)

Init AV = 0

CV = AV/VV

AUCV' = CV

init AUCV = 0

;================================================================================;Mass balance calculations

;================================================================================

{Mass Balance}

Total = DOSE

Calculated = AF + AS + AR + AL + AK+ ALu + AV+ AA + AGI + AMLEE + AMLHA+ AMLHE + AMLAP + AMLM5

ERROR=((Total-Calculated)/Total+1E-30)*100

MASSBBAL=Total-Calculated + 1

;================================================================================;Calculation with model

;calations for graphs supporting hypothesis

PercHE = (AMLHE)*100/DOSE ;percentage of the dose metabolized to HE

PercAP = (AMLAP)*100/DOSE ;percentage of the dose metabolized to AP

PercEE = (AMLEE)*100/DOSE ;percentage of the dose metabolized to EE

PercHA = (AMLHA)*100/DOSE ;percentage of the dose metabolized to HA

PecM5 = (AMLM5)*100/DOSE

PercHES = AMLHES*100/DOSE ;percentage of the dose metabolized to HES

PercHEG = AMLHEG*100/DOSE ;percentage of the dose metabolized to HES

PercOE = AMLOE*100/DOSE ;percentage of the dose metabolized OE

TotalAP = AMLAP

;--------------------------------------------------------------------------------------------------------------------------------------------

;calations for evaluation model performance

HEG_ex=AMLHEG*1000/BW ;amount of HEG formed (nmol/kg bw) to be compared with available in vivo data

HAP_ex=(AMLAP)*1000/BW ;amount of AP formed (nmol/kg bw) to be compared with available in vivo data

; Data: 2011 based on human model of 2009 published in 2012

; Purpose: **Safrole** PBK Final Human Model

; Species: Human

; Compiled by: Ans Punt & Martati Eryana

; Organisation: Wageningen University

;=========================================================

;Physiological parameters

;=========================================================

;Phisiological parameter:Krishnan and Andersen, 2001;Brown et al.,1997

;Tissue Volume

BW = 60 {Kg} ; body weight

VLc = 0.026 ; fraction of liver tissue

VKc = 0.008 ; fraction of kidney tissue

VFc = 0.214 ; fraction of fat tissue

VRc = 0.076-VLc-VKc ; fraction of richly perfused tissue

VSc = 0.81-VFc-VVc ;fraction slowly perfused tissues

;Total fractions 0.91

VVC =0.079; fraction of blood Quick and Shuler 1999 (13.2 mL min-1 k.o.m 0.06) Fraction of blood (see TCDD model cursus)

VF=VFc*BW

VL=VLc*BW

VK=VKc*BW

VR=VRc*BW

VS=VSc*BW

VV=VVc*BW; volume venous blood

;-----------------------------------------------------------------------------------------------------------------------

;Blood flow rates

QCc = 15 {L/hr} ; Info: QC=15*BW^0.74 Reference: Brown, p and Krishnan, p r45

QLc = 0.227 ; Fraction of blood flow to liver

QKc= 0.175 ;fraction of blood flow to kidney

QFc = 0.052 ; Fraction of blood flow to fat

QRc = 0.7-QLC-QKc ; Fraction of blood flow to richly perfused tissue

QSc = 0.3-QFc ; Fraction of blood flow to slowly perfused tissue

; total of fractions = 1

QC=QCc*BW**0.74

QL = QLc*QC {L/hr}

QK=QKc*QC {L/hr}

QF = QFc*QC {L/hr}

QR = QRc*QC {L/hr}

QS = QSc*QC {L/hr}

;========================================================

;Partition Coefficients

;==========================================================

;Safrole

PKS = 6.65 ;kidney/blood partition coefficient

PLS = 6.65 ; liver/blood partition coefficient

PFS =106 ; fat/blood partition coefficient

PRS = 6.65 ; richly perfused tissues/blood partition coefficient

PSS = 4.2 ; slowly perfused tissues/blood partition coefficient

;1'-hydroxysafrole

PLHS = 1.65 ;liver/blood partition coefficient

;========================================================

;Biochemical parameters

;========================================================

;Linear uptake rate (hr-1)

Ka = 1

;-------------------------------------------------------------------------------------------------------------------

;Metabolism liver

{Phase I}

;Scaling factors

S9PL=143; Liver S9 protein yield (mg/gram liver) Pang et al.1985

MPL=32; Liver microsomal protein yield (mg/gram liver) Barter 2007

L=VLC*1000; Liver = 26 (gram/kg BW)

;MPLcyt = 80.7; Liver cytosolic protein yield (mg/g liver) Cubit et al 2011

;metabolites of safrole, unscaled maximum rate of metabolism (nmol min-1 (mg protein)-1)

VmaxLDHSc =0.07 ;DHS=dihydroxysafrole

VmaxLHSc = 0.15 ;HS = 1'-hydroxysafrole

VmaxLHISc = 0.11 ;HIS = 3'hydroxysafrole

VmaxLCHAVc = 0.85 ;CHAV= Dihydroxychavicol

;metabolites of safrole, scaled maximum rate of metabolism (umol hr-1)

VMaxLDHS = VMaxLDHSc/1000*60*MPL*L*BW

VMaxLHS = VMaxLHSc/1000*60*MPL*L*BW

VMaxLHIS = VMaxLHISc/1000*60*MPL*L*BW

VMaxLCHAV = VMaxLCHAVc/1000*60*MPL*L*BW

;metabolites of safrole, affinity constants (umol/L)

KmLDHS =41

KmLHS = 35

KmLHIS = 255

KmLCHAV = 172

{Phase II}

;metabolites of 1'-hydroxysafrole, unscaled maximum rate of metabolism (nmol min-1 (mg protein)-1)

VmaxLHSGc = 0.1 ; HSG = 1'-hydroxysafrole glucuronide

VmaxLHSSc = 0.017 ; HSS = 1'-sulfooxysafrole

VmaxLHSOc = 7.5 ; HSO = 1 oxo safrole

;VmaxLHSOcytc =3.3 ; HSOcyt= 1oxosafrole by cytosolic

;metabolites of 1'-hydroxysafrole, scaled maximum rate of metabolism (umol hr-1)

VMaxLHSG = VmaxLHSGc/1000*60*S9PL*L*BW

VMaxLHSS = VmaxLHSSc/1000*60*S9PL*L*BW

VmaxLHSO = VmaxLHSOc/1000*60*MPL*L*BW

;VmaxLHSOcyt = VmaxLHSOcytc/1000*60*MPLcyt*L*BW

;metabolites of 1'-hydroxysafrole, affinity constants (umol/L)

KmLHSG = 1322

KmLHSS = 3828

KmLHSO = 549

;KmLHSOcyt = 1802

;--------------------------------------------------------------------------------------------------------------

;Metabolism kidney ==> metabolite 3ÓH safrole

;Scaling factors

MPK = 7 ;Kidney microsomal protein yoeld (mg/gram kidney)

K=VKC*1000 ;kidney = 7 (gram/kg BW)

;metabolites of safrole,unscaled maximum rate of metabolism (nmol min-1(mg protein)-1)

VMaxKHISc = 0.03 ;HIS = hydroxyisossafrole

;metabolites of safrole, scaled maximum rate of metabolism (umol hr-1)

VMaxKHIS=VMaxKHISc/1000*60*MPK*K*BW

;metabolite of safrole, affinity constant (umol/L)

KmKHIS = 1054

;---------------------------------------------------------------------------------------------------

;========================================================

;Run settings

;=====================================================

;Molecular weight

MWS = 162.19; Molecular weight safrole

MWHS=178.18; Molecular weight 1'-hydroxysafrole

;Given dose (mg/ kg bw) and oral dose umol/ kg bw} SCF (2002)

GDOSE =0.005 ;(mg/ kg bw} ; GDOSE = given dose

ODOSE = GDOSE*1E-3/MWS*1E6 {umol/ kg bw} ; ODOSE = given dose recalculated to umol/kg bw

DOSE=ODOSE*BW; ; DOSE = umol

;Time

Starttime = 0; in hrs

Stoptime = 120; in hrs

;========================================================

;slowly perfused tissue compartment

;AS = Amount safrole in slowly perfused tissue, umol

AS' = QS*(CA-CVS)

Init AS = 0

CS = AS/VS

CVS = CS/PSS

;-----------------------------------------------------------------------------------------------------------------

;richly perfused tissue compartment

;AR = Amount safrole in richly perfused tissue, umol

AR' = QR*(CA-CVR)

Init AR = 0

CR = AR/VR

CVR = CR/PRS

;----------------------------------------------------------------------------------------------------------------

;fat compartment

;AF = Amount safrole in fat tissue, umol

AF' = QF*(CA-CVF)

Init AF = 0

CF = AF/VF

CVF = CF/PFS

;-----------------------------------------------------------------------------------------------------------

;uptake safrole from GI tract

;AGI = Amount safrole remaining in GI tract (umol)

AGI' = -Ka*AGI

Init AGI = DOSE

;---------------------------------------------------------------------------------------------------------------

;liver compartment

;Safrole

;AL = Amount Safrole in liver tissue, umol

AL' = QL*(CA -CVL)+ Ka*AGI - AMLDHS' - AMLHS' -AMLHIS' -AMLCHAV'

Init AL = 0

CL = AL/VL

CVL = CL/PLS

;AMLDHS = Amount safrole metabolized in liver to dihydroxysafrole (DHS),umol

AMLDHS' = VmaxLDHS*CVL/(KmLDHS + CVL)

init AMLDHS = 0

CLDHS= AMLDHS/VL

;AMLHS = Amount safrole metabolized in liver to 1'-hydroxysafrole (HS),umol

AMLHS' = VmaxLHS*CVL/(KmLHS + CVL)

init AMLHS = 0

FHS=AMLHS/BW

CLHS2 = AMLHS/VL

;AMLHIS = Amount safrole metabolized in liver to 3 Hidroxysafrole (HIS),umol

AMLHIS' =VmaxLHIS*CVL/(KmLHIS+CVL)

init AMLHIS = 0

CLHIS = AMLHIS/VL

;AMLCHAV = Amonut safrole metabolized in liver to Dyhidroxychavicol (CHAV),umol

AMLCHAV'=VmaxLCHAV*CVL/(KmLCHAV+CVL)

init AMLCHAV = 0

CLCHAV = AMLCHAV/VL

;1'-hydroxysafrole sub model

;ALHS = amount 1'-hydroxysafrole in liver tissue, umol

ALHS' = AMLHS'- AMLHSG' -AMLHSO' ; - AMLHSS'

Init ALHS = 0

CLHS = ALHS/VL

CVLHS = CLHS/PLHS

;AMLHSG= amount 1'-hydroxysafrole metabolized in liver to 1'-hydroxysafrole glucurondie (HG),umol

AMLHSG' = VmaxLHSG*CVLHS/(KmLHSG + CVLHS)

init AMLHSG = 0

CLHSG=AMLHSG/VL

FHSG = AMLHSG/BW

;AMLHSS= amount 1'-hydroxysafrole metabolized in liver to safrole 1'sulfate (HSS),umol

AMLHSS' = VMaxLHSS*CVLHS/(CVLHS+KmLHSS)

Init AMLHSS = 0

CLHESS=AMLHSS/VL

FHSS=AMLHSS/BW

;AMLHSO= amount of 1'-hydroxysafrole metabolized in liver to oxo safrole (HSO)

AMLHSO'=VmaxLHSO*CVLHS/(KmLHSO + CVLHS)

init AMLHSO = 0

CLHSO=AMLHSO/VL

FHSO=AMLHSO/BW

;AMLHSOcyt=amount of 1-oh safrole metabolized by cytosolic to oxo safrole (HSO2)

;AMLHSOcyt' = VmaxLHSOcyt*CVLHS/(KmLHSOcyt + CVLHS)

;init AMLHSOcyt = 0

;CLHSOcyt=AMLHSOcyt/VL

;FHSOtot=(AMLHSO+AMLHSOcyt)/BW

;----------------------------------------------------------------------------------------------------------------

;kidney compartment

;AK =amount safrole in KIDNEY tissue,umol

AK'=QK*(CA-CVK)-AMKHIS'

Init AK=0

CK=AK/VK

CVK=CK/PKS

;AMKHIS=amount safrole metabolized in kidney to 3ÓH safrole

AMKHIS'=VmaxKHIS*CVK/(KmKHIS+CVK)

init AMKHIS=0

;------------------------------------------------------------------------------------------------------------------

; venous blood compartment

;CV = Concentration venous blood safrole (umol/L)

AV' = (QF*CVF + QR*CVR + QS*CVS + QL*CVL +QK*CVK)- QC*CV

Init AV = 0

CV = AV/VV

CV2 = (CV*162)/1000

;CVplasma=concentration

;CA= concentration safrole arterial blood (umol/L)

CA=CV

;=========================================================

;Mass balance calculations

;=============================================================

{Mass Balance}

Total = DOSE

Calculated = AF + AS + AR + AL + AK+ AV+ AGI + AMLDHS + AMLHS+ AMLHIS + AMLCHAV + AMKHIS

ERROR=((Total-Calculated)/Total+1E-30)*100

MASSBBAL=Total-Calculated + 1

;========================================================

;Calculation with model

;=======================================================

;calc

PercLDHS=AMLDHS*100/DOSE ;Perc of safrole metabolized to DHS in liver

PercLHS=AMLHS *100/DOSE ;Perc of safrole metabolized to 1oh safrole in liver

PercLHIS=AMLHIS *100/DOSE ;Perc of safrole metabolized to 3Oh safrole in liver

PercLCHAV=AMLCHAV *100/DOSE;Perc of safrole metabolized to Chavicol in liver

PercKHIS=AMKHIS*100/DOSE ;percentage of safrole metabolized to HIS in kidney

;cal

PercLiver=(AMLDHS+AMLHS+AMLHIS+AMLCHAV)*100/DOSE ; perc safrole metabolism in the liver

PercKid=(AMKHIS)*100/DOSE ;perc safrole metabolism in the kidney

;calculations for graphs supporting hypothesis

PercDHS=(AMLDHS)*100/DOSE ;percentage of the dose metabolized to DHS

PercHS = (AMLHS)*100/DOSE ;percentage of the dose metabolized to HS

PercHIS = (AMLHIS+AMKHIS)*100/DOSE ;percentage of the dose metabolized to HIS

PercCHAV = (AMLCHAV)*100/DOSE ;percentage of the dose metabolized to CHAV

PercHSO = (AMLHSO)*100/DOSE ;percentage of the dose metabolized to SO

PercHSS = (AMLHSS)*100/DOSE ;percentage of the dose metabolized to HSS

PercHSG = AMLHSG*100/DOSE ;percentage of dose metabolized to HSG

PercTotalmetabolites = PercDHS + PercHS + PercHIS + PercCHAV

; calculation

;AMLDHSgram=AMLDHS*162

formHSS=AMLHSS/BW ;umol/kg bw

;------------------------------------------------------------------------------------------------------------------------------------------

;calculations for evaluation model performance

HSG_ex=AMLHSG*1000/BW ;amount of HSG formed (nmol/kg bw) to be compared with available in vivo data

AUCCL' = CL

init AUCCL = 0

AUCCLHS' = CLHS

init AUCCLHS = 0

AUCCA' = CA

init AUCCA = 0

AUCCV' = CV

init AUCCV = 0

; Data: 2021 based on human model of Punt et al., 2009

; Purpose: **Methylugenol** PBK Final Human Model

; Species: Human

; Compiled by: Alicia Paini based on the paper by Al-Subeihi et al., 2012

;=======

;Physiological parameters

;======

;Tissue volumes

BW = 60 {Kg}

VLc = 0.026

VFc = 0.214

VAc = 0.02

VVc = 0.059

VRc = 0.076-VLc

VSc = 0.81-VFc-VAc-VVc

VL = VLc*BW {L or Kg}

VF = VFc*BW

VR = VRc*BW

VS = VSc*BW

VA = VAc*BW

VV = VVc*BW

;-----------

;Blood flow rates

QC = 15*BW**0.74 {L/hr}

QLc = 0.227

QFc = 0.052

QRc = 0.70-QLC

QSc = 0.30-QFC

QL = QLc*QC {L/hr}

QF = QFc*QC

QR = QRc*QC

QS = QSc*QC

;=========

;Partition Coefficients

;========

;methyleugenol

PLE = 6.2

PFE = 103

PRE = 6.2

PSE = 3.9

;1'-hydroxymethyleugenol

PLHE = 1.4

;==========

;Biochemical parameters

;==========

;Linear uptake rate (hr-1)

Ka = 1

;-----------------

;Metabolism liver

;Scaling factors

S9PL=143;

MPL=32;

L=VLC*1000;

;metabolites of methyleugenol, unscaled maximum rate of metabolism (nmol min-1 (mg protein)-1)

VmaxLHEc = 1.38

VmaxLAPc = 0.15

VmaxLEEc = 0.66

VmaxLHAc = 0.21

VmaxLM5c = 0.39

VmaxLM6c = 0.10

;metabolites of methyleugenol, scaled maximum rate of metabolism (umol hr-1)

VMaxLHE = VMaxLHEc/1000*60*MPL*L*BW

VMaxLAP = VMaxLAPc/1000*60*MPL*L*BW

VMaxLEE = VMaxLEEc/1000*60*MPL*L*BW

VMaxLHA = VMaxLHAc/1000*60*MPL*L*BW

VMaxLM5 = VMaxLM5c/1000*60*MPL*L*BW

VmaxLM6= VMaxLM6c/1000*60*MPL*L*BW

;metabolites of methyleugenol, affinity constants (umol/L)

KmLHE = 404

KmLAP = 13.6

KmLEE = 23.7

KmLHA = 1097

KmLM5 =161

KmLM6 = 415

;metabolites of 1'-hydroxy methyleugenol, unscaled maximum rate of metabolism (nmol min-1 (mg protein)-1)

VmaxLHEGc = 0.66

VmaxLOEc = 2.1

VmaxLHESc = 0.0009

;metabolites of 1'-hydroxy methyleugenol, scaled maximum rate of metabolism (umol hr-1)

VMaxLHEG = VmaxLHEGc/1000*60*MPL*L*BW

VMaxLOE = VmaxLOEc/1000*60*S9PL*L*BW

VMaxLHES = VmaxLHESc/1000*60*S9PL*L*BW

;metabolites of 1'-hydroxy methyleugenol, affinity constants (umol/L)

KmLHEG = 2393

KmLOE = 1744

KmLHES = 139

;=======

;Run settings

;=======

;Molecular weight

MWS = 178.2; Molecular weight Methyleugenol

MWHS=194.2; Molecular weight 1'-hydroxymethyleugenol

;Given dose (mg/ kg bw) and oral dose umol/ kg bw} SCF (2002)

GDOSE =0.07 ;(mg/ kg bw} ; GDOSE = given dose

ODOSE = GDOSE*1E-3/MWS*1E6 {umol/ kg bw} ; ODOSE = given dose recalculated to umol/kg bw

DOSE=ODOSE*BW; ; DOSE = umol

;Time

Starttime = 0; in hrs

Stoptime = 24; in hrs

;Stoptime = 120; in hrs

;==========

;Dynamics

;===========

;slowly perfused tissue compartment

;AS = Amount methyleugenol in slowly perfused tissue, umol

AS' = QS*(CA-CVS)

Init AS = 0

CS = AS/VS

CVS = CS/PSE

;------------------

;richly perfused tissue compartment

;AR = Amount methyleugenol in richly perfused tissue, umol

AR' = QR*(CA-CVR)

Init AR = 0

CR = AR/VR

CVR = CR/PRE

;-----------------------

;fat compartment

;AF = Amount methyleugenol in fat tissue, umol

AF' = QF*(CA-CVF)

Init AF = 0

CF = AF/VF

CVF = CF/PFE

;--------------------

;uptake methyleugenol from GI tract

;AGI = Amount methyleugenol remaining in GI tract (umol)

AGI' =-Ka*AGI

Init AGI = DOSE

;-------------------

;liver compartment

; methyleugenol

;AL = Amount methyleugenol in liver tissue, umol

AL' = QL*(CA -CVL)+ Ka*AGI - AMLHE' - AMLAP' - AMLEE' -AMLHA'-AMLM5'- AMLM6'

Init AL = 0

CL = AL/VL

CVL = CL/PLE

AUCL' = CL

Init AUCL = 0

;AMLHE=Amount methyleugenol metabolized in liver to 1'-hydroxy methyleugenol (HE)

AMLHE' = VmaxLHE*CVL/(KmLHE + CVL)

Init AMLHE = 0

CMLHE =AMLHE/VL

;AMLAP=Amount methyleugenol metabolized in liver to 4-allylphenol (AP)

AMLAP' = VmaxLAP*CVL/(KmLAP + CVL)

Init AMLAP = 0

CMLAP =AMLAP/VL

;AMLEE=Amount methyleugenol metabolized in liver to methyleugenol -2',3'-oxide (EE)

AMLEE' = VmaxLEE*CVL/(KmLEE + CVL)

Init AMLEE = 0

CMLEE =AMLEE/VL

;AMLHA=Amount methyleugenol metabolized in liver to 3'-hydroxyanethole (HA)

AMLHA' = VmaxLHA*CVL/(KmLHA + CVL)

Init AMLHA = 0

CMLHA =AMLHA/VL

;AMLM5=Amount methyleugenol metabolized in liver to M5 (M5)

AMLM5' = VmaxLM5*CVL/(KmLM5 + CVL)

Init AMLM5 = 0

CMLM5 =AMLM5/VL

;AMLM6=Amount methyleugenol metabolized in liver to M6 (M6)

AMLM6' = VmaxLM6*CVL/(KmLM6 + CVL)

Init AMLM6 = 0

CMLM6 =AMLM6/VL

;1'-hydroxyestragole

;ALHE = amount 1'-hydroxy methyleugenol in liver tissue, umol

ALHE' = AMLHE' - AMLHEG' - AMLHES' - AMLOE'

Init ALHE = 0

CLHE = ALHE/VL

CVLHE = CLHE/PLHE

;AMLHEG= amount 1'-hydroxy methyleugenol metabolized in liver to 1'-hydroxy methyleugenol glucurondie (HEG)

AMLHEG' = VmaxLHEG*CVLHE/(KmLHEG + CVLHE)

Init AMLHEG = 0

CLHEG = AMLHEG/VL

;AMLOE= amount 1'-hydroxy methyleugenol metabolized in liver to 1'-oxo methyleugenol (OE)

AMLOE' = VmaxLOE*CVLHE/(KmLOE + CVLHE)

Init AMLOE = 0

CLOE = AMLOE/VL

;AMLHES= amount 1'-hydroxy methyleugenol metabolized in liver to 1'-sulfooxy methyleugenol (HES)

AMLHES' = VmaxLHES*CVLHE/(KmLHES + CVLHE)

Init AMLHES = 0

CLHES = AMLHES/VL

;----------------------------

; arterial blood compartment

;CA = Concentration arterial blood methyleugenol

CA=CV

;----; venous blood compartment

;CV = Concentration venous blood methyleugenol (umol/L)

AV' = (QF*CVF + QR*CVR + QS*CVS + QL*CVL - QC*CV)

Init AV = 0

CV = AV/VV

AUCV' = CV

Init AUCV = 0

;=====

;Mass balance calculations

;=====

{Mass Balance}

Total = DOSE

Calculated = AF + AS + AR + AL + AV+ AGI + AMLEE + AMLHA+ AMLHE + AMLAP+AMLM5+ AMLM6

ERROR=((Total-Calculated)/Total+1E-30)*100

MASSBBAL=Total-Calculated + 1

;==========

;Calculation with model

;========

;calculations as percentage of the dose metabolized

PercHE = (AMLHE)*100/DOSE

PercAP = (AMLAP)*100/DOSE

PercEE = (AMLEE)*100/DOSE

PercHA = (AMLHA)*100/DOSE

PecM5 = (AMLM5)*100/DOSE

PecM6 = (AMLM6)*100/DOSE

PercHES = (AMLHES)*100/DOSE

PercHEG = (AMLHEG)*100/DOSE

PercOE = (AMLOE)*100/DOSE
