# Supplementary material for: Assessment of the predictive capacity of a physiologically based kinetic model using a read-across approach
Source: Comput Toxicol. 2021 May;18:100159. doi: 10.1016/j.comtox.2021.100159 (PMC8130669; doi:10.1016/j.comtox.2021.100159)
Supplement: Supplementary data 3 [file mmc3.docx]

**Annex III**

**Evaluation of the application of the Estragole model to make predictions for Methyleugenol - PBK model predictions using data from analogues – using the OECD PBK model template.**

This document was developed as part of the OECD PBK model Guidance Document work and linked to this paper as supporting information for transparency in model reporting and evaluation.

Contents

[Part I. PBK model reporting template 1](#_Toc50207490)

[Part II Checklist for model evaluation 13](#_Toc50207491)

[Part III Final Evaluation 16](#_Toc50207492)

## Part I. PBK model reporting template

A. Name of model

PBK model predictions using data from analogues

B. Contact details

Alicia Paini (1), Sunil Kulkarni (2), Judith Madden (3), and Andrew Worth(1)

1) EC Joint Research Centre; 2) Health Canada; 3) Liverpool John Moore University

C. Summary of model characterisation, development, validation, and potential applicability

The purpose is to provide means to evaluate a PBK model for chemicals for which no in vivo reference data are available (toxicity, ADME , TK parameters). This will help to establish safe levels (for humans) for both cases of acute (e.g. acute reference dose) or sub-chronic/chronic (e.g. acceptable daily intake) for regulated compounds (for REACH it is the derived no effect level [DNEL]) or tolerable daily intake for contaminants in food. The approach can be applied for prioritisation of chemicals, and for hazard characterisation, to set a point of departure.

The present study provides an example of PBK model evaluation using (structural) analogues of a chemical under assessment with no in vivo data available for the target chemical. On the basis of the results available, it was concluded that using data from one chemical source (estragole) for read across to a target chemical (methyleugenol) is a reasonable approach for a preliminary hazard characterisation, in the absence of in vivo data available to fully validate a methyleugenol model. Furthermore, the application of a PBK model that takes into account the kinetics of the chemical understudy reduces the uncertainties of the A, D, M, and E characteristics of the chemical

**Disclaimer:** In the present case study we report information for two well-known, previously studied chemicals, with valid PBK models, as a proof-of-principle. The chemicals under study are estragole and methyleugenol. Methylegenol will be considered, hypothetically, as a data–poor chemical requiring PBK model validation against human in vivo data.

The PBK model code for estragole in humans (Punt et al., 2009, 2016); was used with the following alterations:

1. The MW for parent and hydroxy-metabolite for estragole (known to be the active metabolite) were substituted with the values for methyleugenol as were the partition coefficients, predicted using the approach of Brown et al and the chemical specific LogKow.

2. In addition to the above changes, the in vitro Vmax and Km, values measured, for the formation of several metabolites formed in phase I and phase II were also substituted from those relevant to estragole of those relevant for methyleugenol. These values were available for estragole and methyleugenol (Al-Subeihi et al., (2012) and by Punt et al., (2009; 2016), see table A.2).

We report the information and validation steps for the PBK model of the source chemical estragole and information on the parameters for the target chemical, methyleugenol.

D. Model characterisation (modelling workflow)

Step 1 – Scope and purpose of the model (problem formulation)

Evaluation of PBK models for chemicals that lack data for parametrisation and to assess model validity. In this case study, we need to simulate time and dose concentration response curve of methyleugenol, to assess human exposure after food ingestion – diet – for risk assessment.

Using the Tanimoto index of structural similarity (according to the calculations of the OECD QSAR Toolbox 4.4) estragole and methyleugenol have a similarity score of 0.70.

**Premise**

Methyleugenol lacks of a valid PBK model, and in vivo data are not available*. A PBK model for estragole was developed to assess human exposure and was validated. Data is used from one chemical source (estragole) for read across to a target chemical (methyleugenol).

(*hypothetically posited to enable a proof-of-principle analysis)

Step 2 – Model conceptualisation (model structure, mathematical representation)

A PBK model was developed and evaluated against in vivo human data to describe the relative importance of bioactivation and detoxification of estragole in humans at different oral dose levels using in vitro data (Punt et al., 2009). The model used in this case study was developed for estragole and then applied to methyleugenol. (For methyleugenol a PBK model does already exist (Al-Subeihi et al., 2012) and in this case could be used to validate the proof-of-principle. Following the mode of action of the two chemicals being investigated, a similar PBK model structure can be applied to both, focusing on the target organ the liver, but also including lungs and kidney since metabolism also occurs in these organs. The rate of formation of metabolites in lungs and kidney is generally negligible, however they are included in the model to account for wider variation that may be present in a population where some individuals may have higher metabolism in these other organs.

The ADME characteristics of estragole were captured by the following model structure and equations:

- 1. Organs involved in determining the chemical’s ADME profile were identified
  2. The model structure is reported in (figure A.2); the model consists of several compartments representing liver, fat tissue, and other rapidly/richly or slowly/poorly perfused tissues) connected via the systemic circulation.
  3. The Berkley Madonna PBK model code is available at the end of this document.
  4. Metabolism was considered in the target organ (liver) and was based on the proposed biotransformation pathways of estragole.
  5. Punt et al., (2009; 2016) developed and parameterised the PBK model for estragole using in vitro data and human physiological information; this model was evaluated on in vivo data as described in Punt et al., 2009.

As they are similar in terms of mechanism of toxic action action and similar in structure, the estragole PBK code was used to derive a model for methyleugenol (see figure 1), since the Tanimoto index of structural similarity estragole and methyleugenol have a similarity score of 0.70.


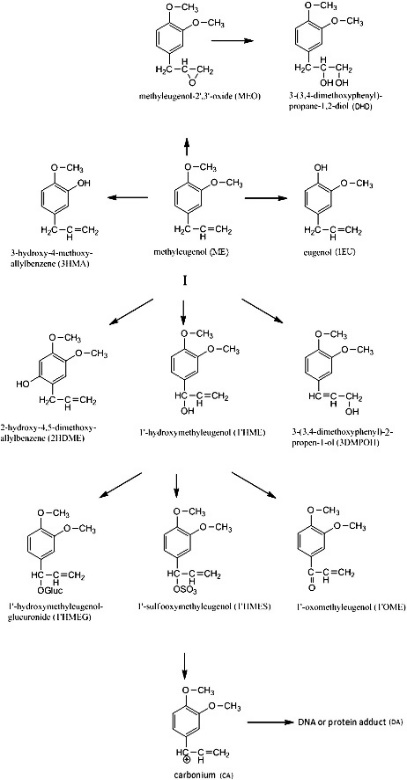

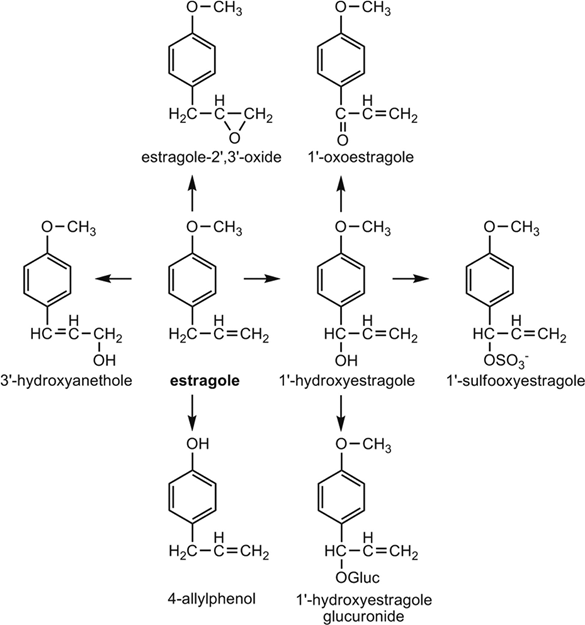


Figure 1. Proposed metabolic pathways of the alkenylbenzene methyleugenol, taken from Al-Subeihi et al., (2012), and estragole, Punt et al (2009). The two chemicals show same mode of action in metabolism, the difference can be the amount formed of each metabolite

Since similar MOA action and similar in structure the estragole code was applied for methyleugenol.

The PBK Model structure reported in figure 2 is built by separating in different compartments liver, lung and kidney and lumping remaining organs as either rapidly/richly perfused or slowly/poorly perfused. This division was done in accordance with the capacity of the organ to form metabolites of the parent chemical under investigation and based on the mode of action of the chemicals.


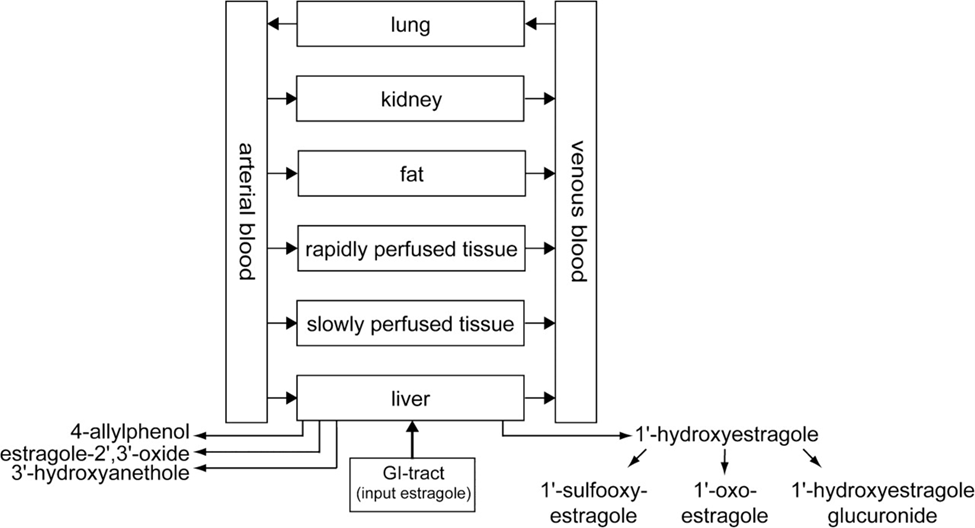


Fig. 2. Human PBK model structure used to describe mathematically the chemical’s (estragole’s) fate in the body, taken from Punt et al., 2009.

.

The PBK model for methyleugenol was based on the proposed biotransformation pathways of estragole as shown in figure 1. The difference in metabolism between estragole and methyleugenol is that methyleugenol has an additional two metabolites formed, 3-(3,4 – dimethoxyphenyl)-2-propen-1-ol (3-DMPOH) and 2-hydroxy-4,5-dimethoxyallylbenzene (2HDME), that do not appear in estragole pathway so far published in the literature. However, the main pathway leading to adverse outcome, DNA adduct binding, is via hydroxylation, this path is similar path for both chemicals. Using the human PBK models for estragole, a comparison could be made between the model predictions of formation of the 1'-hydroxy metabolite (figure 1). However, for phase II metabolite formation, both the 1′-sulfoxy metabolite and glucuronide gave the predictions where different using this approach versus the original methyleugenol model.

A summary list of assumptions on the PBK model are reported:

1. First-order kinetics was used to describe the uptake of methyleugenol from the gastrointestinal (GI) tract assuming a direct uptake by the liver with an absorption rate constant (*k* a) of 1.0 h−1, which is based on the fast and complete absorption of the structurally related alkenylbenzene estragole from the GI tract.
2. Liver was found to be the only target organ for metabolism of estragole and methyleugenol, however, the lack of metabolism in other organs could also be due to low sensitivity of analytics.
3. Because of similar structure and similar MoA to other alkenylbenzenes a read across approach could be applied. However, estragole lacks two metabolites that methyleugenol forms, this could also be due to low sensitivity of analytics.
4. Metabolism plays a crucial role; it is known that the toxic MoA of these chemicals is driven by the hydrolysis pathway with formation of the 1’-hydroxy-metabolite. Here the PBK models for the parent compounds are compared, however toxicity is driven by their metabolites. All alkenyl benzene chemicals will react through the same metabolic pathways, but probably with different rate of formation, via Phase I CYPs and Phase II enzymes.

Step 3 – Model parameterisation (parameter estimation and analysis)

Physiological parameterisation of the PBK model Table 1. Reports the physiological parameters used in the PBK model (see section i).

Table 2. Reports the physicochemical and kinetic parameters used in the PBK model.

In vitro parameterisation of the PBK model for methyleugenol (Al-Subeihi et al., 2012) these parameters were established by

- Microsomal metabolism of methyleugenol using mixed pooled human S9 fraction.
- Glucuronidation of 1′-hydroxy methyleugenol to 1′-hydroxy methyleugenol glucuronide.
- Oxidation of 1′-hydroxymethyleugenol to 1′-oxo methyleugenol.
- Sulfonation of 1′-hydroxy methyleugenol to 1′-sulfoxy methyleugenol using Paps as a cofactor and GSH as scavenger due to instability of the 1′-sulfoxymetabolite in an aqueous environment.
- Identification and quantification of metabolism of methyleugenol and 1′-hydroxy methyleugenol by UPLC.
- Determination of kinetic constants

Parameters used in the PBK model for methyleugenol in human

- Physicochemical proprieties, online, Chemspider or EPA Chemistry Dashboard.
- Physiological parameters (Brown et al. 1997)
- Tissue: blood partition coefficients (DeJongh et al.1997) based on Log *K* ow values for methyleugenol and 1′-hydroxy methyleugenol

Step 4 – Computer implementation (solving the equations)

The equations were coded and numerically integrated in Berkeley Madonna 8.3.18 (Macey and Oster, UC Berkeley, CA) using Rosenbrock’s algorithm for stiff systems. PBK models in human liver were run for 72h, because that would be the time for total clearance of methyleugenol in human tissues after one dose.

The code was run using Berkeley Madonna version 8.3.23.0, on august 2018 (the code is available at the end of this document).

The Code of the model is reported in the appendix of this template

Step 5 – Model Performance

Model performance was assessed by checking the estragole PBK model:

1. The mass balance equation was used to evaluate the model description/stability.
2. A local sensitivity analysis was performed (figure 3), normalized sensitivity coefficients (SCs) were determined using the following equation:

SC = (C′ - C)/(P′ - P) × (P/C)

where C is the initial value of the model output, C′ is the modified value of the model output resulting from an increase in parameter value, P is the initial parameter value, and P′ represents the modified parameter value. An increase of 5 % in parameter values was used to analyze the effect of a change in parameter on the formation of 1′-hydroxy metabolite and 1′-sulfoxymetabolite (expressed as a percentage of the dose). Each parameter was analyzed individually, while the other parameters were kept at their initial value.

Comparison was made of the PBK model-based prediction of bioactivation of methyleugenol to the PBK model-based predictions for bioactivation of the structurally related compound estragole.

1. The human PBK model was validate by comparing historical data available for excretion of the glucuronidation in humans. In addition the model was also compared to the rodent PBK model that was validated using in vivo data.

We believe that once the PBK model for estragole passed the model performance and gained confidence it can be applied in a read across manner to incorporate the data of methyleugenol.

Step 6 – Model Documentation

The following models were previously published in peer review journals (Punt, A. et al., 2009. Al-Subeihi et la., 2011. The read across approach described within this case study will be published soon (Paini et al., in preparation).

E. Identification of uncertainties

The methyleugenol predictions were run using a “valid” PBK model built for estragole, results were compared to the estragole outcomes, but also compared to the “valid” methyleugenol model (Al-Subeihi et al., 2011).

Model structure

The structure of the PBK model was simplified to capture the only organs and metabolic pathways that were relevant for the source chemical estragole. The authors report that only the liver, and to a minor capacity also lungs and kidney, showed a significant metabolic rate of exchange compared to the other organs, metabolism in kidney was neglected. The PBK Model structure reported in figure 2 is built by separating in different compartments liver, lung and kidney and lumping up in rapidly/richly perfused and slowly perfused the remaining organs. This division was done in accordance of their –organ- capacity in metabolising – biotransformation of the parent/chemicals understudy and based on the mode of action of the chemicals

Input parameters

1. Although a well-established protocol to assess the formation of metabolite(s), the application of mixed gender human pooled S9 could be restricted to a few donors and therefore not be reflective of the overall variability in the population; variability in metabolic pathways may be much greater with more donors.
2. The in vitro Vmax and Km can carry additional uncertainty based on human variability (in ADME or metabolism) (although pooled batches are often used) and differences in sample preparation and sensitivity in the analytical (method) to measure the samples, in also quantification of concentrations of the chemical and its metabolite(s).
3. Sensitivity analysis (OAT) was conducted on the estragole PBK model, to identify the most sensitive parameters, which were identified as the Vmax and Km Kinetic constant. Figure 3.


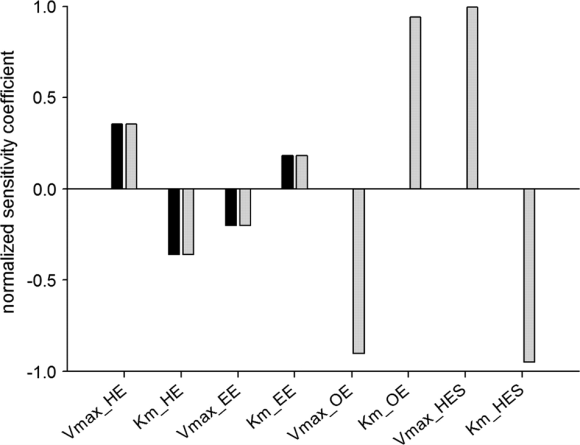


Figure 3. Sensitivity of the predicted formation of 1′-hydroxymetabolite (black bars) and 1′-sulfooxymetabolite (gray bars) to different model parameters. The Vmax and Km correspond to the maximum rate of formation and the Michaelis-Menten constant for the formation of the different metabolites in the liver for estragole: 1′-hydroxyestragole (HE), estragole-2′,3′-oxide (EE), 1′-oxoestragole (OE) and 1′-sulfooxyestragole (HES), taken from Punt et al., 2009 .

Model output

Uncertainties in output: Variability in model output (over prediction and under prediction, depending on the metabolite) between using the read-across model versus the original model. Due to model input data and model structure. Sensitivity analysis was conducted on the estragole PBK model, to identify the most sensitive parameters, which were identified as the Vmax and Km constant for the formation of the different metabolites in the liver: 1′-hydroxyestragole (HE), and 1′-sulfooxyestragole (HES). A change in this value can lead to a change in model output.

Other uncertainties (e.g. model developed for different substance and/or purpose)

Other uncertainties: Although in this example we used two chemicals for which we had a valid PBK model, still some uncertainties can be found in the process proposed.

**Overall evaluation of uncertainties**: On the basis of the results available it was concluded that the using data and available model code from one chemical (source) to read across to the target is a reasonable approach for a preliminary risk assessment, where in vivo data are not available. Furthermore, the application of a PBK model that takes into account the kinetics of the chemical understudy reduces the uncertainties of A, D, M, and E.

F. Model implementation details

- software (version no): The code was run using Berkeley Madonna version 8.3.23.0, on August 2018 (the code is available at the end of this document).
- availability of code: Yes, reported below in appendix.
- software verification / qualification : was done by peer reviewed publication.

G. Peer engagement (input/review)

Was not performed but the model was published in peer reviewed journals, see reference list

H. Parameter tables

Table 1. Reports the physiological parameters used in the PBK model.

| Parameter name | Value | Unit |
| --- | --- | --- |
| Body weight | 60 | Kg |
| Cardiac output | 15 | (L/hr-1kg) |
| Fractional blood flow to fat - QFC | 0.052 |  |
| Fractional blood flow to liver -QLC | 0.227 |  |
| Fractional blood flow to richly perfused tissues QRC | 0.70-QLC |  |
| Fractional blood flow to slowly perfused tissues - QSC | 0.30-QFC |  |
| Fraction fat tissue VFC | 0.214 |  |
| Fraction of liver - VLC | 0.026 |  |
| Fraction richly perfused tissue - VRC | 0.076-VLC |  |
| Fraction slowly perfused tissue - VSC | 0.81-VFC-VBC |  |
| Fraction of blood Quick and Shuler 1999 | VBC = 0.079 |  |

Table 2. Reports the physicochemical and kinetic parameters used in the PBK model.

|  | MW/ Hydroxyl metabolite MW | Vmax/Km | PC |
| --- | --- | --- | --- |
| Estragole source  Punt et al., (2009; 2016) | 148.2/164.2 | {Phase I}  VmaxLHEc = 0.7; HE = 1'-hydroxyestragole, Max rate of metabolism (nmol min-1(mg protein)-1)  VmaxLAPc = 0.4; AP = 4-allylphenol,  VmaxLEEc = 0.9; EE = estragole-2',3'-oxide,  VmaxLHAc = 1.4; HA = 3'-hydroxyanethole,  KmLHE = 21;Affinity constant (umol/L)  KmLAP = 290  KmLEE = 83  KmLHA = 350  {Phase II}  VmaxLHEGc = 0.29; HEG = 1'-hydroxyestragoleglucuronide, Max rate of metabolism (nmol min-1(mg protein)-1)  VmaxLHESc = 0.0074; AHE = 1'-sulfooxyestragole,  VmaxLOEc = 5  KmLHEG = 708; Affinity constant (umol/L)  KmLHES = 727  KmLOE = 345 | PFE = 105; Fat/blood partition coefficient  PRE = 6.5;Richly perfused tissues/blood partition coefficient  PSE = 4.0;Slowly perfused tissues/blood partition coefficient  PLE = 6.5;Liver/blood partition coefficient  {1'-hydroxy-met}  PLHE = 1.6; Liver/blood partition coefficient |
| Methyleugenol target  (Al-Subeihi et al., 2012) | 178.2/194.2 | {Phase I}  VmaxLHEc = 1.38; HE = 1'-hydroxyestragole, Max rate of metabolism (nmol min-1(mg protein)-1)  VmaxLAPc = 0.15; AP = 4-allylphenol,  VmaxLEEc = 0.66; EE = estragole-2',3'-oxide,  VmaxLHAc = 0.39; HA = 3'-hydroxyanethole,  KmLHE = 404;Affinity constant (umol/L)  KmLAP = 13.6  KmLEE = 23.7  KmLHA =161  {Phase II}  VmaxLHEGc =  0.66; HEG = 1'-hydroxyestragoleglucuronide, Max rate of metabolism (nmol min-1(mg protein)-1)  VmaxLHESc = 0.0009; AHE = 1'-sulfooxyestragole,  VmaxLOEc = 2.1  KmLHEG = 2393; affinity constant (umol/L)  KmLHES = 139  KmLOE = 1774 | PFE = 103; Fat/blood partition coefficient  PRE =6.2;Richly perfused tissues/blood partition coefficient  PSE = 3.9;Slowly perfused tissues/blood partition coefficient  PLE = 6.2;Liver/blood partition coefficient  {1'-hydroxy-met}  PLHE = 1.4; Liver/blood partition coefficient |

#

Strategy for reducing overall uncertainty (excluding generation of data based on new animal test)

Generate data using volunteer human subjects.

Application of methyleugenol in vitro metabolic parameters

To make the read-across approach more robust - apply the approach using more analogues which have valid model codes and check the results.

Based on the local sensitivity analysis performed Vmax and km were the parameters identified that could influence the model output most significantly. The following table shows the uncertainty/variability of the parameter estimates.

| **Variability/Uncertainty in the Parameter Estimates** | | |  |  |
| --- | --- | --- | --- | --- |
|  | | High | Medium | Low |
| **SENSITIVITY** | High | Variability in Vmax and Km from in vitro studies from different donors or pooled |  |  |
|  | Medium |  | Analytics - Measurement of Vmax and Km |  |
|  | Low |  |  | Extrapolation from in vitro to in vivo of Vmax and Km |

| **Variability/Uncertainty in the Parameter Estimates** | | |  |  |
| --- | --- | --- | --- | --- |
|  | | High | Medium | Low |
| **SENSITIVITY** | High |  |  |  |
|  | Medium |  |  |  |
|  | Low |  |  | PC and Physic chemical proprieties from literature and QSARs |

In conclusion the present study provides an example of PBK model evaluation using (structural) analogues of a chemical under assessment with no in vivo data available for the target chemical.

On the basis of the results available, it was concluded that using data from one chemical source (estragole) for read across to methyleugenol is a reasonable approach for a preliminary hazard characterisation, in the absence of in vivo data available to fully validate a methyleugenol model. Furthermore, the application of a PBK model that takes into account the kinetics of the chemical understudy reduces the uncertainties of A, D, M, and E.

I. References and background information

- publications

1. Punt, A. Paini, A., Boersma, M.G Freidig, A.P. Delatour T., Scholz G., Schilter B., van Bladeren P.J., Rietjens I.M.C.M.(2009) Use of physiologically based biokinetic (PBBK) modeling to study estragole bioactivation and detoxification in humans as compared to male rats Toxicological Sciences, 110 (2009), pp. 255-269
2. Al-Subeihi, A.A Spenkelink, B. Rachmawati N., Boersma M.G, Punt A., Vervoort J., van Bladeren P. J., Rietjens I. M.C.M., (2011) Physiologically based biokinetic model of bioactivation and detoxification of the alkenylbenzene methyleugenol in rat, Toxicology in Vitro, Volume 25, Issue 1, 2011, Pages 267-285.

- code

**Estragole PBK model code written in Berkeley Madonna (Punt et al., 2009 and 2016)**

{Punt et al., 2009; Physiological Parameters: Krishnan and Andersen, 2001; Brown et al., 1997}

---------------------------------------

;Physiological parameters

;Tissue volumes

BW = 60 {Kg} ; body weight human

VLc = 0.026 ; fraction of liver tissue

VKc = 0.004 ; fraction of kidney tissue

VLuc = 0.008 ; fraction of lung tissue

VFc = 0.214 ; fraction of fat tissue

VAc = 0.02 ; fraction of arterial blood: 0.079*1/4

VVc = 0.059 ; fraction of venous blood: 0.079*3/4

VRc = 0.08-VLc-VLuc-VKc ; fraction of richly perfused tissue

VSc = 0.836-VFc-VAc-VVc ; Fraction of blood flow to slowly perfused tissue

; total of fractions = 0.916

VL = VLc*BW {L or Kg}

VLu= VLuc*BW

VK = VKc*BW

VF = VFc*BW

VR = VRc*BW

VS = VSc*BW

VA = VAc*BW

VV = VVc*BW

;Blood flow rates

QCc = 15 ; Info: QC=15*BW^0.74 Reference: Brown

QC = QCc*BW**0.74 {L/hr} ; Info: QC=15*BW^0.74 Reference: Brown

QLc = 0.227 ; Fraction of blood flow to liver

QKc = 0.175 ; Fraction of blood flow to kidney

QFc = 0.052 ; Fraction of blood flow to fat

QRc = 0.70-QLC-QKC ; Fraction of blood flow to richly perfused tissue

QSc = 0.30-QFC ; Fraction of blood flow to slowly perfused tissue

; total of fractions = 1

QL = QLc*QC {L/hr}

QK = QKc*QC {L/hr}

QF = QFc*QC {L/hr}

QR = QRc*QC {L/hr}

QS = QSc*QC {L/hr}

;estragole (E)

PLE = 6.5 ; liver/blood partition coefficient

PLuE=6.5 ; lung/blood partition coefficient

PKE=6.5 ; kidney/blood partition coefficient

PFE = 105 ; fat/blood partition coefficient

PRE = 6.5 ; richly perfused tissues/blood partition coefficient

PSE = 4.1 ; slowly perfused tissues/blood partition coefficient

;1'-hydroxyestragole (HE)

PLHE = 1.6 ;liver/blood partition coefficient

;Linear uptake rate (hr-1)

Ka = 1

;Metabolism liver

;Scaling factors

S9PL=143 ; Liver S9 protein yield (mg/gram liver) Pang et al.1985

MPL=32 ; Liver microsomal protein yield (mg/gram liver) Atio et al. 1976

L=VLC*1000 ; Liver = 26 (gram/kg BW)

;metabolites of estragole, unscaled maximum rate of metabolism (nmol min-1 (mg protein)-1)

VmaxLHEc = 0.73 ;HE = 1'-hydroxyestragole,

VmaxLAPc = 0.38 ;AP = 4-allylphenol

VmaxLEEc = 0.85 ;EE = estragole-2',3'-oxide

VmaxLHAc = 1.35; ;HA = 3'-hydroxyanethole

VmaxLM5c = 0.18 ;M5 = metabolites 5

;metabolites of estragole, scaled maximum rate of metabolism (umol hr-1)

VMaxLHE = VMaxLHEc/1000*60*MPL*L*BW

VMaxLAP = VMaxLAPc/1000*60*MPL*L*BW

VMaxLEE = VMaxLEEc/1000*60*MPL*L*BW

VMaxLHA = VMaxLHAc/1000*60*MPL*L*BW

VMaxLM5 = VMaxLM5c/1000*60*MPL*L*BW

;metabolites of estragole, affinity constants (umol/L)

KmLHE = 21

KmLAP = 290

KmLEE = 83

KmLHA = 350

KmLM5 = 618

;metabolites of 1'-hydroxyestragole, unscaled maximum rate of metabolism (nmol min-1 (mg protein)-1)

VmaxLHEGc = 0.3 ; HEG = 1'-hydroxyestragole glucuronide

VmaxLOEc = 4.9 ; OE= 1'-oxoestragole

VmaxLHESc = 0.007 ; HES = 1'-sulfooxyestragole

;metabolites of 1'-hydroxyestragole, scaled maximum rate of metabolism (umol hr-1)

VMaxLHEG = VmaxLHEGc/1000*60*MPL*L*BW

VMaxLOE = VmaxLOEc/1000*60*S9PL*L*BW

VMaxLHES = VmaxLHESc/1000*60*S9PL*L*BW

;metabolites of 1'-hydroxyestragole, affinity constants (umol/L)

KmLHEG = 708

KmLOE = 354

KmLHES = 727

;Molecular weight

MWE = 148.2; Molecular weight estragole

MWHE= 164.2; Molecular weight 1'-hydroxyestragole

;Given dose (mg/ kg bw) and oral dose umol/ kg bw}

GDOSE = 0.07 {mg/ kg bw} ; GDOSE = given dose

ODOSE = (GDOSE*1E-3)/MWE*1E6 {umol/ kg bw} ; ODOSE = given dose recalculated to umol/kg bw

DOSE=ODOSE*BW; ; DOSE = umol

;Time

Starttime = 0; in hrs

Stoptime = 24; in hrs

;AS = Amount estragole in slowly perfused tissue, umol

AS' = QS*(CA-CVS)

Init AS = 0

CS = AS/VS

CVS = CS/PSE

;AR = Amount estragole in richly perfused tissue, umol

AR' = QR*(CA-CVR)

Init AR = 0

CR = AR/VR

CVR = CR/PRE

;AF = Amount estragole in fat tissue, umol

AF' = QF*(CA-CVF)

Init AF = 0

CF = AF/VF

CVF = CF/PFE

;AGI = Amount estragole remaining in GI tract (umol)

AGI' =-Ka*AGI

Init AGI = DOSE

;AL = Amount Estragole in liver tissue, umol

AL' = QL*(CA -CVL)+ Ka*AGI - AMLHE' - AMLAP' - AMLEE' -AMLHA'

Init AL = 0

CL = AL/VL

CVL = CL/PLE

AUCL' = CL

init AUCL = 0

;AMLHE=Amount estragole metabolized in liver to 1'-hydroxyestragole (HE)

AMLHE' = VmaxLHE*CVL/(KmLHE + CVL)

init AMLHE = 0

CMLHE =AMLHE/VL

CMBWHE =AMLHE/BW*1000 ;nmol/kg bw

;AMLAP=Amount estragole metabolized in liver to 4-allylphenol (AP)

AMLAP' = VmaxLAP*CVL/(KmLAP + CVL)

init AMLAP = 0

CMLAP =AMLAP/VL

CMBWAP =AMLAP/BW*1000 ;nmol/ kg bw

;AMLEE=Amount estragole metabolized in liver to estragole-2',3'-oxide (EE)

AMLEE' = VmaxLEE*CVL/(KmLEE + CVL)

init AMLEE = 0

CMLEE =AMLEE/VL

CMBWEE =AMLEE/BW*1000 ;nmol/kg bw

;AMLHA=Amount estragole metabolized in liver to 3'-hydroxyanethole (HA)

AMLHA' = VmaxLHA*CVL/(KmLHA + CVL)

init AMLHA = 0

CMLHA =AMLHA/VL

CMBWHA =AMLHA/BW*1000

;AMLM5=Amount estragole metabolized in liver to M5 (M5)

AMLM5' = VmaxLM5*CVL/(KmLM5 + CVL)

Init AMLM5 = 0

CMLM5 =AMLM5/VL

;ALHE = amount 1'-hydroxyestragole in liver tissue, umol

ALHE' = AMLHE' - AMLHEG' - AMLHES' - AMLOE'

Init ALHE = 0

CLHE = ALHE/VL

CVLHE = CLHE/PLHE

AUCLHE' = CLHE

init AUCLHE = 0

;AMLHEG= amount 1'-hydroxyestragole metabolized in liver to 1'-hydroxyestragole glucurondie (HEG)

AMLHEG' = VmaxLHEG*CVLHE/(KmLHEG + CVLHE)

init AMLHEG = 0

CLHEG = AMLHEG/VL ;nmol/g liver (umol/kg liver)

CBWHEG = AMLHEG/BW*1000 ;nmol/kg bw

;AMLOE= amount 1'-hydroxyestragole metabolized in liver to 1'-oxoestragole (OE)

AMLOE' = VmaxLOE*CVLHE/(KmLOE + CVLHE)

init AMLOE = 0

CLOE = AMLOE/VL

CBWOE = AMLOE/BW*1000

;AMLHES= amount 1'-hydroxyestragole metabolized in liver to 1'-sulfooxyestragole (HES)

AMLHES' = VmaxLHES*CVLHE/(KmLHES + CVLHE)

init AMLHES = 0

CLHES = AMLHES/VL

CBWHES = (AMLHES/BW)*1000 ;nmol/kg bw

;AK = amount estragole in liver tissue, umol

AK' = QK*(CA -CVK)

Init AK = 0

CK = AK/VK

CVK = CK/PKE

;ALu = amount estragole in lung tissue, umol

ALu' = QC*(CV-CALu)

Init ALu = 0

CLu = ALu/VLu

CALu = CLu/PLuE

;CA = Concentration arterial blood estragole

AA' = QC*(CALu-CA);

Init AA = 0

CA = AA/VA

;CV = Concentration venous blood estragole (umol/L)

AV' = (QF*CVF + QR*CVR + QS*CVS + QL*CVL + QK*CVK - QC*CV)

Init AV = 0

CV = AV/VV

AUCV' = CV

init AUCV = 0

{Mass Balance}

Total = DOSE

Calculated = AF + AS + AR + AL + AK+ ALu + AV+ AA + AGI + AMLEE + AMLHA+ AMLHE + AMLAP + AMLM5

ERROR=((Total-Calculated)/Total+1E-30)*100

MASSBBAL=Total-Calculated + 1

## Part II Checklist for model evaluation

| **PBK Model Evaluation Checklist** | **Checklist assessment** | **Comments** |
| --- | --- | --- |
| **Name of the PBK model (as in the reporting template)** | PBK model predictions using data from analogues | |
| **Model developer and contact details** | Alicia Paini (1), Sunil Kulkarni (2), Judith Madden (3), and Andrew Worth(1)  1) EC Joint Research Centre; 2) Health Canada; 3) Liverpool John Moore University | |
| **Name of person reviewing and contact details** | **L. Rossi** | |
| **Date of checklist assessment** | **04/04/2020** | |
| **A.   Context/Implementation** |  |  |
| **A.1. Regulatory Purpose** |  |  |
| 1.    What is the acceptable degree of confidence/uncertainty (e.g. high, medium or low) for the envisaged application (e.g. priority setting, screening, full assessment?) | **High** |  |
| 2.    Is the degree of confidence/uncertainty in application of the PBK model for the envisaged purpose greater or less than that for other assessment options (e.g. reliance on PBK model and *in vitro* data vs. no experimental data)? | **High** |  |
| **A.2. Documentation** |  |  |
| 3.    Is the model documentation adequate, i.e. does it address the essential content of model reporting template, including the following: | **Yes** |  |
| - Clear indication of the chemical, or chemicals, to which the model is applicable? | **Yes** |  |
| - Is the model being applied for the same scientific purpose as it was developed, or has it been repurposed somehow? | **Yes** |  |
| - Model assumptions? | **Yes** |  |
| - Graphical representation of the proposed mode of action, if known? | **Yes** |  |
| - Graphical representation of the conceptual model? | **Yes** |  |
| - Supporting tabulation for parameters (names, meanings, values, mean and standard deviations, units and sources)? | **Yes** |  |
| - Relevance and reliability of model parameters? | **No** |  |
| - Uncertainty and sensitivity analysis? | **Yes** |  |
| - Mathematical equations? | **No** |  |
| - PBK model code? | **Yes** |  |
| - Software algorithm to run the PBK model code? | **Yes** |  |
| - Qualification of PBK software platform? | **No** |  |
| **A.3 Software Implementation and Verification** |  |  |
| 4.    Does the model code express the mathematical model? | **Yes** | **the code represents the chemical MoA** |
| 5.    Is the model code devoid of syntactic and mathematical errors? | **Yes** |  |
| 6.    Are the units of input and output parameters correct? | **Yes** |  |
| 7.    Is the chemical mass balance respected at all times? | **Yes** |  |
| 8.    Is the cardiac output equal to the sum of blood flow rates to the tissue compartments? | **Yes** |  |
| 9. Is the sum total of tissue volumes equal to total body volume? | **No** | **Equals to 0.91** |
| 10. Is the mathematical solver a well-established algorithm? | **Yes** |  |
| 11. Does the mathematical solver converge on a solution without numerical error? | **Yes** |  |
| 12. Has the PBK modelling platform been subjected to a verification process (for a different use, for instance, in the pharmaceutical domain)? | **No** |  |
| **A.4 Peer engagement (input/review)** |  |  |
| 13.  Has the model been used previously for a regulatory purpose? | **Yes** | **EMA used the estragole PBK model predictions to evaluate the chemical** |
| - Is prior peer engagement in the development and review of the model sufficient to support the envisaged application? | **Yes** |  |
| - Is additional review required? Peer engagement includes input/review by experts on specific aspects of model development, individual reviews of the model by experts, or collective reviews by peer review panels. Availability of the comments and tracking of revisions to the model in response to peer input contributes to increased confidence in the model for potential application. | **No** |  |
| **B.   Assessment of Model Validity** |  |  |
| **B.1 Biological Basis (Model Structure and Parameters)** |  |  |
| 14.  Is the model consistent with known biology? | **Yes** |  |
| - Is the biological basis for the model structure provided? | **Yes** |  |
| - Is the complexity of the model structure appropriate to address the regulatory application? | **Yes** |  |
| - Are assumptions concerning the model structure and parameters clearly stated and justified? | **Yes** |  |
| - Is the choice of values for physiological parameters justified? | **Yes** |  |
| - Is the choice of methods used to estimate chemical-specific ADME parameters justified? | **Yes** |  |
| - Saturable kinetics | **Yes** | **By means of Km and Vmax** |
| **B.2 Theoretical Basis of Model Equations** |  |  |
| 15. Are the underlying equations based on established theories, .e.g. Michaelis-Menten kinetics, Fick’s laws of diffusion? | **Yes** |  |
| - In the case of PBK models for particles, does the model take into consideration the properties of particles, e.g. particle size ranges, (poor) solubility, aggregation, partitioning and diffusion/sedimentation behaviour? | **NA** |  |
| **B.3. Reliability of input parameters** |  |  |
| 16.  Has the uncertainty (individual variability, experimental reproducibility and reliability) in the input parameters been characterised? | **Partially** | **In the measured values we have std & varibility** |
| **B.4. Uncertainty and Sensitivity Analysis** |  |  |
| 17.  Has the impact of uncertainty (individual variability, experimental reproducibility and reliability) in the parameters on the chosen dose metric been estimated? | **Yes by OTA SA** |  |
| - Local sensitivity analysis? | **Yes** |  |
| - Global sensitivity analysis? | **No** |  |
| **18**.  Is confidence in influential input parameter estimates (i.e., based on comparison of uncertainty and sensitivity) reasonable (within expected values; similar to those of analogues) in view of the intended application? | **Yes** |  |
| **B.5. Goodness-of-Fit and Predictivity** |  |  |
| 19.  For PBK models for which there are sufficient *in vivo* data for the chemical of interest: |  |  |
| - Suitability as analogue (chemical and biological similarity) been assessed? | **Yes** |  |
| - Reliable estimation of chosen dose metric for analogue? | **Yes** |  |
| - In general is the biological Variability of *in vivo* reference data (from analogue) established? | **Yes** |  |

## Part III Final Evaluation

Overall conclusion on mode evaluation for the intended application

**HIGH**

**NONE**

**Model**

**simulations**

**of data; predictivity**

**Biological**

**basis**

Model reproduces consistently the shape of time and dose course profiles for the analogue.

The model is built using the current knowledge on the chemical MoA, and is parameterised using in vitro measured data that are well established

The model validity is established for the analogue.

.

**LEVEL OF CONFIDENCE**

**Variability/ Uncertainty in Parameter Analysis; Global Sensitivity Analysis**

No GLOBAL sensitivity analyses were carried out, only local SA performed to establish which parameter is most sensitive.

The model is built using the current knowledge on the source chemical MoA (estragole), and is parametrise using in vitro metabolic measured data (for the target methyleugenol) using well established assays. The PBK model reproduces consistently the shape of time and dose course profiles for the analogue. The PBK model validity is established for source chemical, thus we assume that the PBK model is valid also for the target chemicals (methyleugenol).

Recommendation is to gain more confidence in dose metric prediction probably a GSA should have been performed. However, by running an OAT SA we identified the key parameter that can perturb the model output most significantly.
